# Regulation of chromatin accessibility and gene expression in the developing hippocampal primordium by LIM-HD transcription factor LHX2

**DOI:** 10.1371/journal.pgen.1010874

**Published:** 2023-08-18

**Authors:** Varun Suresh, Bhavana Muralidharan, Saurabh J. Pradhan, Mahima Bose, Leora D’Souza, Arpan Parichha, Puli Chandramouli Reddy, Sanjeev Galande, Shubha Tole

**Affiliations:** 1 Department of Biological Sciences, Tata Institute of Fundamental Research, Mumbai, India; 2 Institute for Stem Cell Science and Regenerative Medicine, Bangalore, India; 3 Chromatin Biology and Epigenetics Laboratory, Biology department, Indian Institute of Science Education and Research Pune, India; 4 Department of Life Sciences, Shiv Nadar Institution of Eminence, Gautam Buddha Nagar, Delhi NCR, India; University of California San Diego, UNITED STATES

## Abstract

In the mammalian cerebral cortex, the hippocampal primordium (Hcp) occupies a discrete position in the dorsal telencephalic neuroepithelium adjacent to the neocortical primordium (Ncp). We examined transcriptomic and chromatin-level features that distinguish the Hcp from the Ncp in the mouse during the early neurogenic period, embryonic day (E)12.5. ATAC-seq revealed that the Hcp was more accessible than the Ncp at this stage. Motif analysis of the differentially accessible loci in these tissues revealed LHX2 as a candidate transcription factor for modulating gene regulatory networks (GRNs). We analyzed LHX2 occupancy profiles and compared these with transcriptomic data from control and *Lhx2* mutant Hcp and Ncp at E12.5. Our results revealed that LHX2 directly regulates distinct genes in the Hcp and Ncp within a set of common pathways that control fundamental aspects of development namely pluripotency, axon pathfinding, Wnt, and Hippo signaling. Loss of *Lhx2* caused a decrease in accessibility, specifically in hippocampal chromatin, suggesting that this factor may play a unique role in hippocampal development. We identified 14 genes that were preferentially enriched in the Hcp, for which LHX2 regulates both chromatin accessibility and mRNA expression, which have not thus far been examined in hippocampal development. Together, these results provide mechanistic insight into how LHX2 function in the Hcp may contribute to the process by which the hippocampus acquires features distinct from the neocortex.

## Author summary

The brain performs an array of functions via functionally specialized structures. We examined how the hippocampus, a structure that is critical for learning and memory, acquires distinct molecular features from an adjacent structure, the neocortex, which processes different functions. As development proceeds, nuclear DNA, which is packaged into DNA-protein complexes called chromatin, is acted upon by regulatory factors that modulate its accessibility to the molecular machinery that produces messenger RNA (mRNA). Measurements of chromatin accessibility and quantification of gene-specific mRNA production, called gene expression profiling, are key tools to uncover early steps in how stem cells give rise to daughter cells with diverse functional capabilities. We found that the early embryonic hippocampal chromatin was significantly more accessible than that of the neocortex. We identified a regulatory protein, LHX2 that maintains this accessibility selectively in the hippocampus, and also controls the expression of distinct sets of genes in the hippocampus and the neocortex. Finally, we identified 14 LHX2 target genes that are important candidates for regulating hippocampal development. Our integrated multiomics approach offers an insight into how the hippocampus and the neocortex develop their unique neuronal compositions which underlie their specialized functions.

## Introduction

The mammalian hippocampus arises from the dorsal telencephalic neuroepithelium that lies adjacent to that of the neocortical primordium (Ncp). The hippocampal primordium (Hcp) contains apical progenitors in the ventricular zone, intermediate progenitors in the subventricular zone, postmitotic neurons in the overlying cortical plate, and Cajal-Retzius cells in the marginal zone, similar to the cellular composition of the Ncp. The proliferation and differentiation of these cell types are regulated by a common set of transcription factors (TFs) e.g. PAX6, SOX2, and FOXG1 [1–3] in both structures. The acquisition of Hcp regional identity is expected to involve the regulation of chromatin both in terms of accessibility and histone modifications. Therefore, we analyzed these features in chromatin obtained from the Hcp and the Ncp at embryonic day (E) 12.5 in the mouse, when the neuroepithelium is predominantly proliferative and neurogenesis is in its early stages. The binding motif of transcription factor LHX2 emerged as a candidate in differentially accessible loci in the Hcp and Ncp chromatin.

LHX2 is a well-established regulator of distinct phenomena in early Hcp and Ncp development (reviewed in [4]). Loss of *Lhx2* prior to E10.5 causes the Hcp and Ncp to transform into the hem and antihem, respectively [5–7]. Between E10.5 and E11.5, loss of *Lhx2* causes the Ncp to acquire characteristics of the paleocortical primordium [8]. Loss of *Lhx2* from E11.5 causes a range of phenotypes that have been well-characterized: early cell cycle exit of Ncp and Hcp progenitors leading to thinning of the superficial layers of the neocortex [9]; loss of the corpus callosum [10]; profoundly deficient thalamocortical innervation of the neocortex accompanied by the reduced electrical activity of subplate neurons [11,12]; reduction in layer 6 TBR1+ neurons and increase in layer 5 FEZF2+/CTIP2+ neurons [13]; drastic shrinkage of the hippocampus [14]. These studies motivated a chromatin-level analysis of LHX2 function in the Ncp and Hcp. We hypothesized that Lhx2 may participate in unique gene regulatory networks (GRNs) in the Hcp and Ncp by comparing chromatin accessibility, histone modifications, and transcriptomic changes in the cortex-specific *Lhx2* conditional mutant Ncp and Hcp. We report that Lhx2 regulates unique genes in each tissue that map to four major developmental/signaling pathways. Furthermore, we found loss of *Lhx2* leads to a decrease in chromatin accessibility, specifically in the Hcp, suggesting it is a major regulator of the Hcp chromatin state, and may control a cascade of processes that promote a distinct identity to the developing hippocampus.

## Results

**Fig 1:**
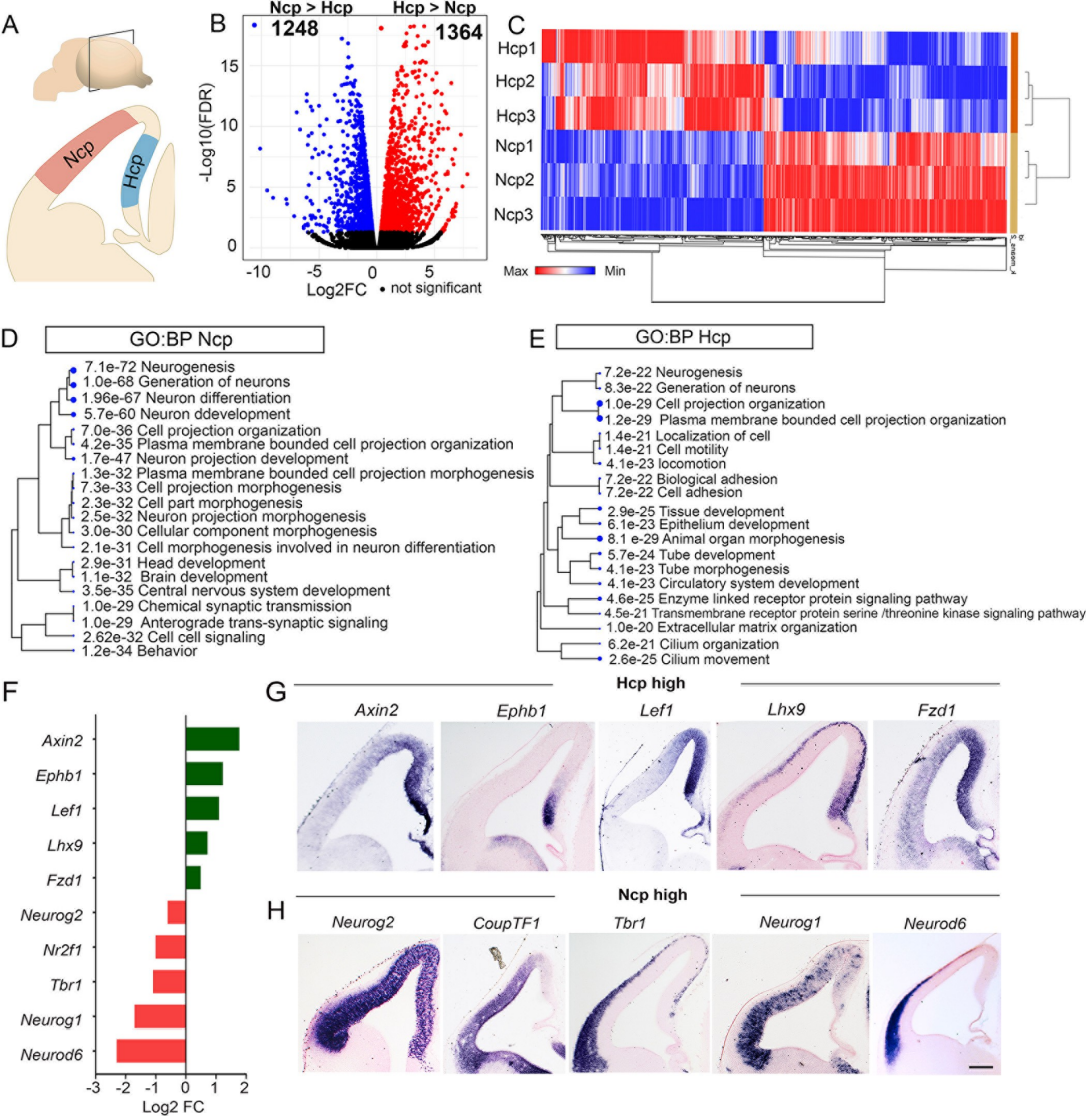
Transcriptomic analysis of the wild type E12.5 Ncp and Hcp. (A) Schematic representation of the E12.5 mouse brain. (B) A volcano plot comparing Ncp and Hcp mRNA expression identifies preferentially enriched genes in the Ncp (1248) and the Hcp (1364). (C) A heatmap of the top 100 enriched genes in each tissue type; color bar: blue (low expression), red (high expression), clustering method: K means. (D, E) A tree plot depicting the top GO Biological Processes (GO: BPs) from (B). (F) Bar plot of selected differentially expressed genes between Ncp and Hcp. (G, H). *In situ* hybridization for selected genes identified in (B).

We isolated the Hcp and Ncp from E12.5 brains, as shown in the diagram, from intact hemispheres for all analyses (Fig 1A) [15]. Both tissues contain apical+basal progenitors, postmitotic neurons, and Cajal-Retzius cells at this stage. The progenitors in each tissue are actively producing neurons [15,16], although the Ncp tissue is likely to contain more postmitotic neurons than the Hcp tissue at its lateral extreme (Fig 1H). Fundamental regulators of progenitor proliferation, PAX6, FOXG1, SOX2, and TBR2, are expressed in both tissues at this stage [17–19]. To examine transcriptomic differences between the Ncp and Hcp, we performed RNA-seq (Fig 1B and S1 Table). We identified 1248 Ncp (Ncp>Hcp; p < 0.05) enriched and 1364 Hcp enriched genes (Hcp>Ncp; p < 0.05, Fig 1B and 1C).

As expected, the top enriched Biological Processes (BPs) in both primordia span a range of neurodevelopmental phenomena, including neurogenesis, generation of neurons, and plasma membrane-bounded cell projection organization. However, each tissue is enriched with a distinct set of genes for each process, suggesting that although the Ncp and Hcp share similar cellular compositions consisting of apical and basal progenitors, newborn neurons, and Cajal-Retzius cells, the regulatory process that govern development in these tissues may be different (Fig 1C and 1D and S1 Table). Based on our RNA-seq results, we performed RNA in-situ hybridizations of select genes. Among the Hcp > Ncp genes were Wnt signaling components such as *Fzd1*, *Lef1*, and *Axin2*, as well as previously reported Hcp markers such as *Lhx9* and *Ephb1* (Fig 1F–1H [20]). Several TFs were also identified to be differentially enriched (111 Ncp > Hcp; 94 Ncp < Hcp) that are known or putative regulators of forebrain development (Fig 1F–1H1 and S1 Table). We also identified BPs enriched in only one tissue, such as cell-cell signaling and synaptic transmission in the Ncp and cell adhesion, extracellular matrix organization, and cilium organization in the Hcp (Fig 1D and 1E). Analysis of the top KEGG pathways displayed a similar pattern, with the Wnt signaling pathway being common to both primordia, but axon guidance, synaptic vesicle cycle, cAMP and MAPK signaling pathway being enriched in the Ncp, whereas TGF-beta signaling, Hippo pathway, and pathways regulating pluripotency of stem cells being enriched in the Hcp (S2A and S2B Fig). Overall our results revealed major transcriptomic differences between the Ncp and Hcp, suggesting that distinct GRNs are operational in each of these two tissue types.

**Fig 2:**
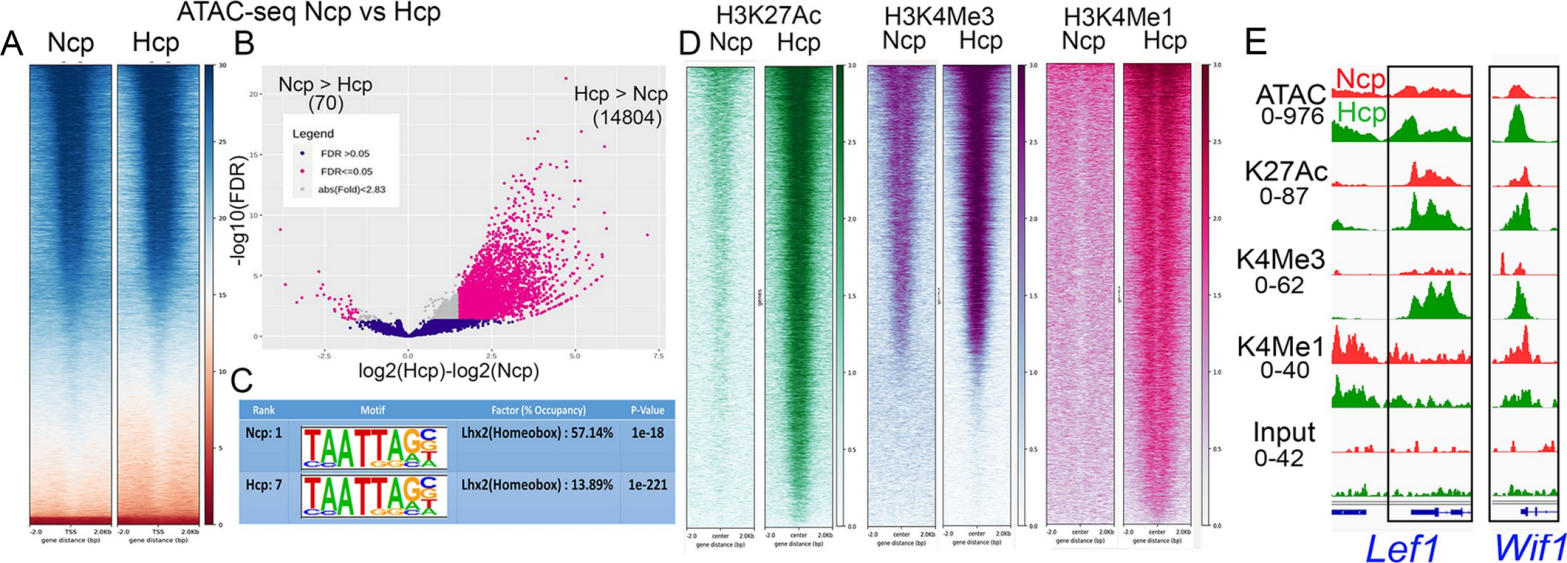
Chromatin accessibility comparison of the E12.5 wild type Ncp and Hcp. (A) A heatmap comparing open chromatin in the Ncp and Hcp. (B) Differential accessibility analysis shows 14804 loci (9508 genes) to be preferentially open in the Hcp and 70 loci (64 genes) to be more open in the Ncp. (C) Motif analysis of the differentially open loci identified in (B) reveals LHX2 among the top candidates. (D) Heat maps display greater active histone modifications on the 14804 loci identified as more open in the Hcp. (E) Genomic loci corresponding to the *Lef1* and *Wif1* loci demonstrating the correspondence between the open chromatin and activating histone marks in the Ncp (red) and Hcp (green). Black boxes mark regions enriched in open chromatin in the Hcp that align with one or more histone modifications. The numbers indicate the maximum peak height for each pair of (Hcp/Ncp) tracks.

**Fig 3:**
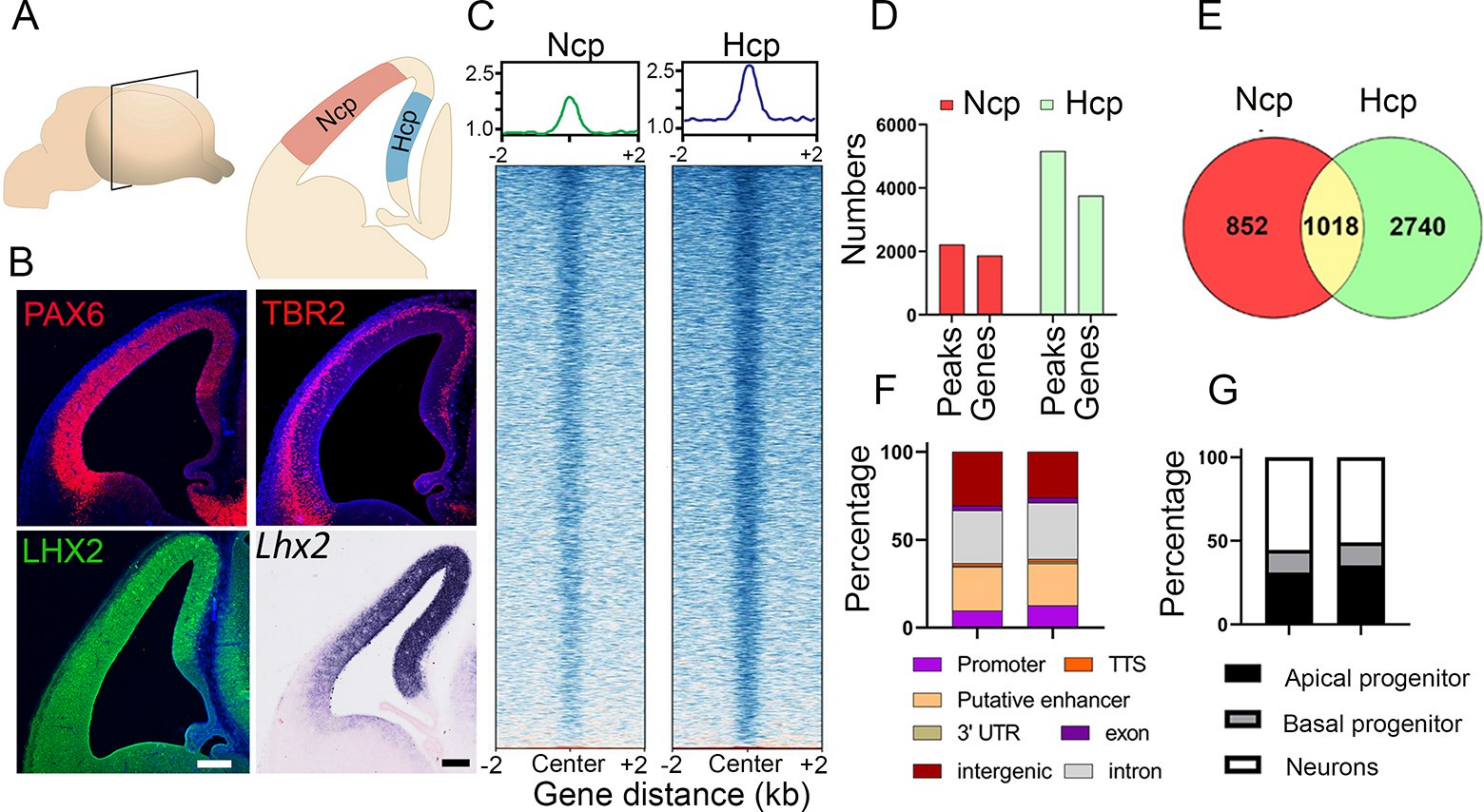
LHX2 occupancy in the E12.5 mouse neocortical and hippocampal primordia (Ncp and Hcp, respectively). (A) Schematic representation of the E12.5 mouse brain. (B) Progenitor markers PAX6, LHX2, and TBR2 immunostaining/*in situ* hybridization (*Lhx2)* in the Ncp and Hcp. (C-G) LHX2 ChIP-seq data in the Ncp and Hcp. Plots of PePr peak-called regions in the Ncp and Hcp show TF LHX2 occupancy in each tissue. Only statistically significant peaks were used for further analysis (p-value 0.0001 and fold change over input: cut off >10 fold) (C); The number of LHX2 occupancy peaks and associated number of genes (D); common genes occupied by LHX2 between the Ncp and Hcp (E); Percentage of LHX2 occupancy peaks categorized by type of genomic region (F); LHX2-occupied genes enriched in different cortical cell types identified by gene enrichment profiles in [28](G). The scale bars in B are 100 μm.

The distinct transcriptomic profile of the E12.5 Ncp and Hcp motivated a comparative analysis of chromatin accessibility using ATAC-seq (assay for transposase-accessible chromatin sequencing) and identified similar numbers of accessible loci in both tissues (>100,000; Fig 2A). However, the Hcp was more accessible in 14804 loci (differentially accessible regions; DARs) which mapped to 9580 DAR-associated genes (DAGs), while 70 DARs (64 DAGs) were enriched in the Ncp (Fig 2B; FDR < 0.05, fold change > 1.5, [21]). Consistent with this, active histone marks H3K27Ac, H3K4Me3, and H3K4Me1 displayed greater occupancy in the 14804 Hcp>Ncp DARs (Fig 2D). To identify potential regulators of change in chromatin state, we investigated transcription factor-binding motifs in the DARs. Of the top 10 motifs, LIM-HD transcription factor LHX2 emerged as a factor of interest (Figs 2C and S2C-S2E) because it was the only one expressed at E12.5 in both tissues ([5], Fig 3B), and known to have stage-specific and cell type-specific roles in the early development of the neocortex and hippocampus [4]. Examples of Hcp-enriched genes, *Lef1* and *Wif1* [20,22], display greater chromatin accessibility in the Hcp than in the Ncp, and these chromatin regions also display greater occupancy of active histone marks (Fig 2E).

Since LHX2 emerged as a common factor in the motif analysis of the DARs in the Ncp and Hcp, we investigated its genomic occupancy in these tissues. We performed ChIP-seq to examine LHX2 occupancy in these tissues and identified 2222 binding sites mapping to 1870 genes in the Ncp and 5166 binding sites mapping to 3758 genes in the Hcp. Of these, 1018 genes were common to the Ncp and Hcp (Fig 3C–3E). Binding on promoters was limited to 9–12% in both tissues, whereas regions such as introns, putative enhancers, and intergenic regions accounted for the majority of the occupancy loci (Figs 3F and S3 and S3 Table). These results suggest that global LHX2 function may be linked to occupancy not only at the TSS/promoter but also in intronic and intergenic regions as well as 5’ or 3’ UTRs as shown across multiple systems (S3H Fig [13,23–25]).

At E12.5, both tissues contain a mix of progenitors (apical and basal) and newly produced postmitotic neurons. *Lhx2* is expressed in each of these populations (Fig 3B, [26–28]. We compared the LHX2-occupied genes with those known to be enriched in apical progenitors/basal progenitors/postmitotic neurons in a single-cell RNA-seq (scRNA-seq) dataset of Ncp tissue [28]. Although no such dataset is available for Hcp tissue, the similarities in cell type composition permitted a comparison and revealed that LHX2-bound genes corresponded to those enriched in progenitors (31% of LHX2-occupied genes in Ncp and 35% in Hcp) as well as those enriched in postmitotic neurons (55% of LHX2-occupied genes in Ncp and 50% in Hcp, Fig 3G), consistent with its diverse roles in multiple aspects of telencephalic development [4].

**Fig 4:**
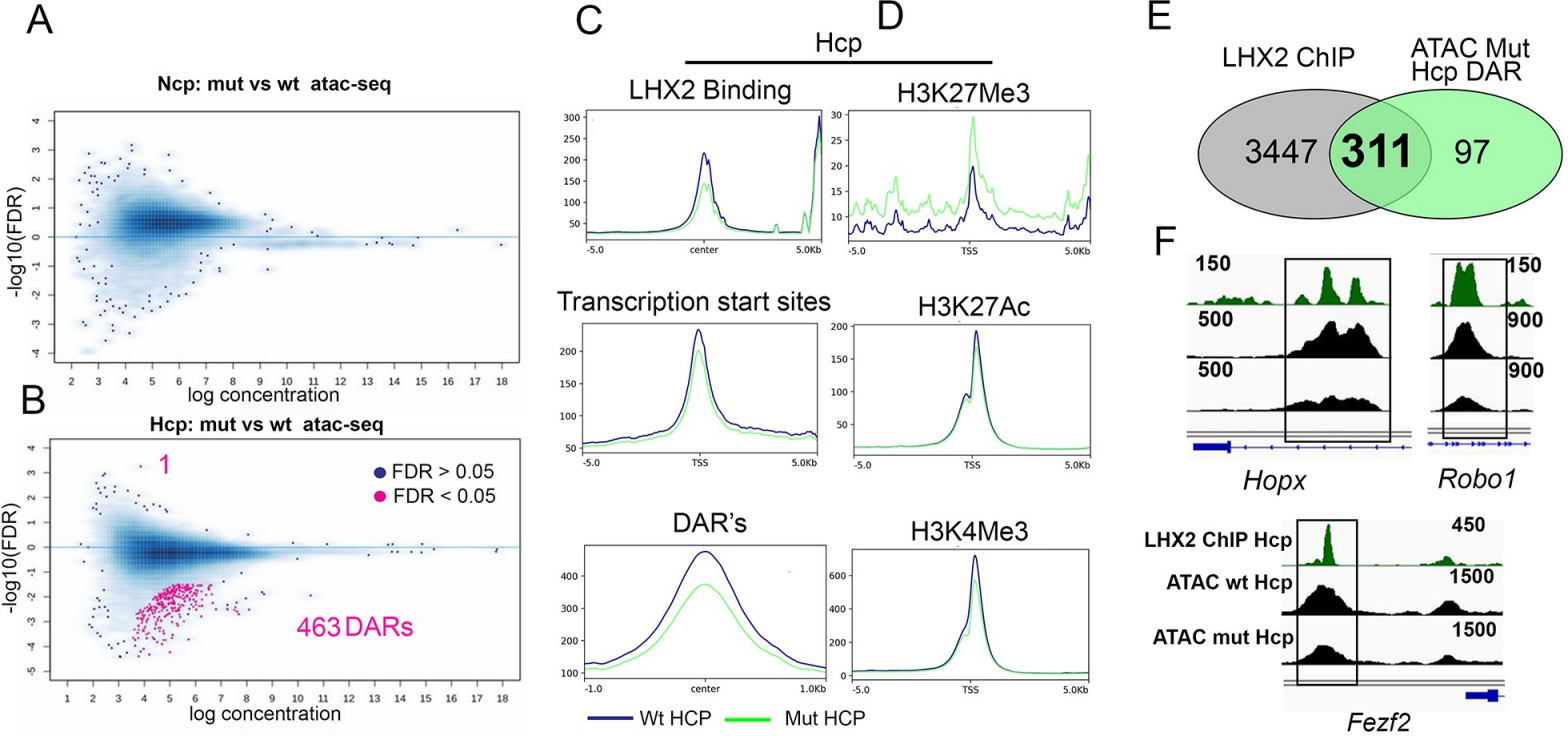
Chromatin accessibility changes upon loss of *Lhx2* in the Ncp and Hcp. (A, B) Scatter plots comparing control versus *Lhx2* mutant chromatin identify loci for which the global accessibility has changed upon loss of *Lhx2* in the Ncp (0) and Hcp (463+1 DARs which map to 405+1 DAGs). (C) Plot profile comparisons of wild type and mutant chromatin in the Hcp showing that the mutant chromatin is less accessible at LHX2 binding sites, TSS, and regions identified in Fig 2B to be differentially accessible (DARs) between wtNcp and wtHcp. (D) Histone modification profiles in the Hcp focusing on the TSS reveal that the loss of *Lhx2* appears to be associated with an increase in the repressive mark H3K27Me3, a reduction in the mark H3K4Me3, and no apparent change in H3K27Ac. (E) A Venn diagram illustrates the majority of the down-regulated DARs are associated with an LHX2 binding peak in the Hcp. (F) Examples of genomic loci showing LHX2 binding regions at which chromatin accessibility is decreased upon loss of *Lhx2* (also see S6D Fig).

For subsequent analysis, we individually focused on the Ncp and Hcp, comparing wild-type and *Lhx2* mutant tissue in each case. The LHX2 occupancy profile motivated an examination of how this factor may regulate chromatin accessibility and histone modifications associated with active or repressed loci. Therefore, we induced conditional loss of *Lhx2* using a dorsal telencephalon-specific driver, Emx1Cre [29]. This Cre line acts from E11.5 [9], ideally suited to examine the potentially immediate effects of loss of *Lhx2* in the Ncp and Hcp by E12.5. A comparison of accessibility of wild-type (wt) and *Lhx2* mutant (mut) Ncp and Hcp respectively, was performed using the DESeq2 package [21]. Loss of *Lhx2* did not alter global chromatin accessibility significantly in Ncp (Fig 4A). In contrast, the loss of *Lhx2* caused a striking reduction in accessibility in the Hcp at 463 DARs (405 Differentially Accessible Genes, DAGs) and an increase in accessibility in 1 DAR (1 DAG; Fig 4B). Since these data were a result of pairwise locus comparisons across the genome, we classified the comparisons based on TSS, DARs, and LHX2 binding region (LHX2BR) and compared data for these categories (Fig 4C). In the Hcp, all three categories displayed greater accessibility in the wild type than in the mutant tissue. Consistent with this, ChIP-seq for the repressive H3K27Me3 mark revealed a sharp increase in occupancy in the mutant. The active H3K27Ac and H3K4Me3 marks displayed little or no change in occupancy upon the loss of *Lhx2* (Fig 4D). We correlated the DARs (463 up +1 down) that exhibit a change in accessibility upon loss of *Lhx2* with LHX2 genomic occupancy in the Hcp. 360 of these DARs mapped to 311 genes that showed LHX2 occupancy. The majority of these are in intronic or intergenic regions (S3I Fig). IGV tracks of some examples of these loci, *Fezf2*, *Robo1*, *and Hopx*, revealed loss of open chromatin at or near the site of LHX2 occupancy (Fig 4E and 4F), suggesting a role for this factor in maintaining specific loci open in the Hcp. In summary, the loss of *Lhx2* in the Hcp renders the chromatin less accessible. This feature is not seen in the Ncp, suggesting that the regulation of chromatin accessibility may be an Hcp-specific function of LHX2.

**Fig 5:**
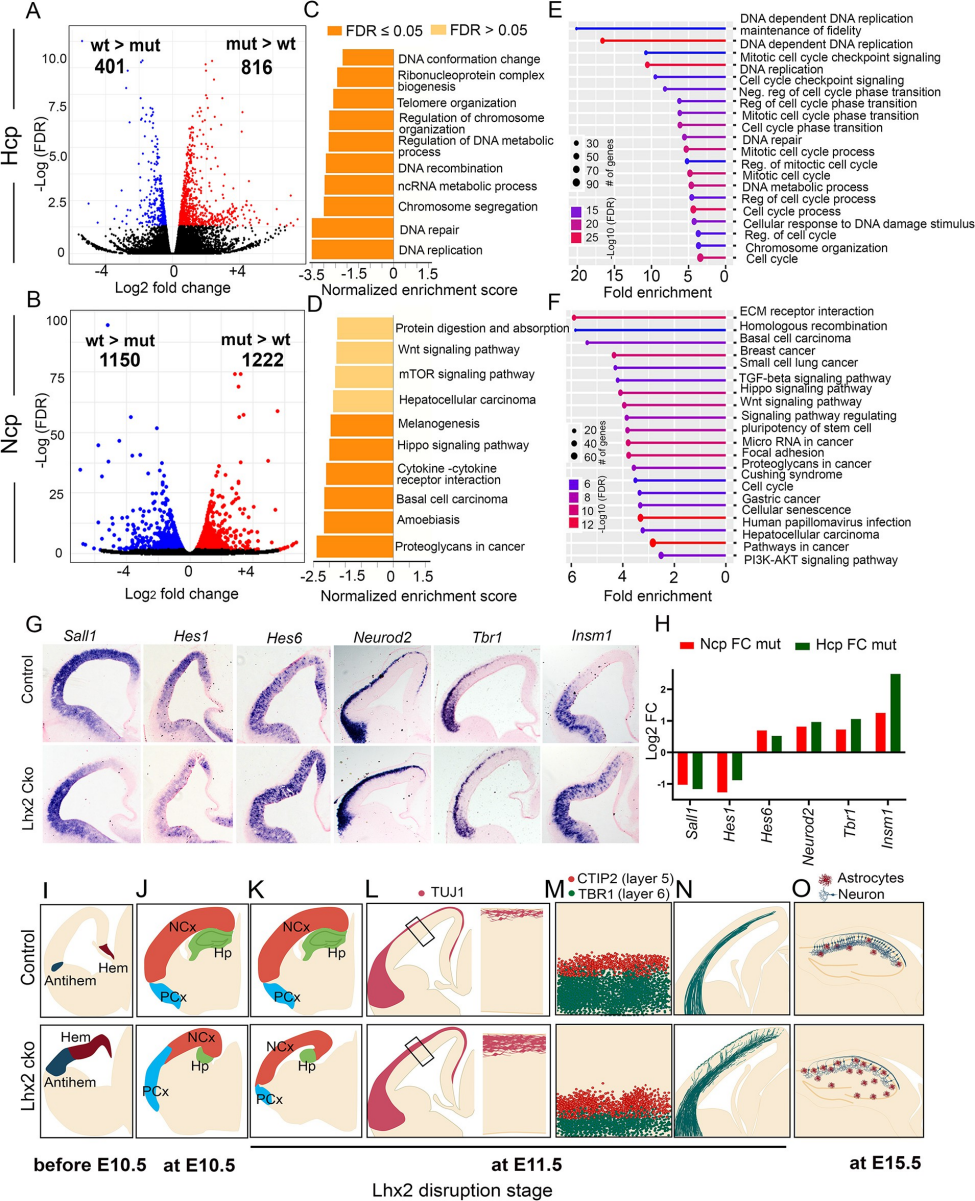
Loss of *Lhx2* causes distinct patterns of transcriptomic dysregulation in the Ncp and Hcp. (A, B) Volcano plots displaying genes dysregulated in each tissue upon loss of *Lhx2*. (C-F) Gene Ontology Biological Processes (GO: BP) corresponding to genes downregulated in Hcp (C, E) and Ncp (D, F). (C, D) shows the GSEA analysis and (E, F) shows the overrepresentation test of the GO: BPs. The corresponding upregulated gene analysis is in S4 Fig. (G) mRNA *in situ* hybridization at E12.5 for some dysregulated genes in the *Emx1Cre*::*Lhx2cko*. (H) Corresponding bar plots of the mRNA fold changes from the RNA-seq data. (I-O) Schematics summarizing loss of *Lhx2* phenotypes. (I-K, O) are partially modified from[4]. (I) Disruption before E10.5 causes the dorsal telencephalic primordium to take on the fate of the hem and the antihem [5,6,39]. (J) Disruption from E10.5 results in shrinkage of the neocortical primordium (Ncp) and expansion of the paleocortical primordium [8]. (K-N) Disruption at E11.5 causes the Ncp and Hcp progenitors to exit the cell cycle early, resulting in the dramatic shrinking of both structures (K, L; [13,32]) due to premature neurogenesis; a perturbation of cell fate such that TBR1+ layer 6 neurons are reduced in number, and CTIP2+ layer 5 neurons are increased in number (M; [13]); thalamocortical axons (green fibers) prematurely grow into the cortical plate due to a deficit in the subplate (N; [11]). (O) *Lhx2* disruption at E15.5 in hippocampal progenitors results in premature gliogenesis during the neurogenic period [60].

A major functional consequence of changes in chromatin accessibility is the alteration of gene expression [30,31]. The *Lhx2* mutant phenotype has been extensively characterized in the neocortex and hippocampus after inducing conditional loss of function at different stages (reviewed in [4]; Fig 5I–5O). We sought to identify the GRNs that were affected by the loss of *Lhx2* in the Ncp and the Hcp by analyzing RNA-seq data. In the Ncp, 2372 differentially expressed genes (DEGs) were identified by comparing wild-type Ncp and mutant Ncp datasets. These consisted of 1150 DEGs that were downregulated (wtNcp > mutNcp) and 1222 that were upregulated (mutNcp > wtNcp) in the mutant Ncp (FDR <0.05; Fig 5B, 5G and 5H). In the Hcp, 1217 DEGs were identified by comparing wild-type Hcp and mutant Hcp datasets. These consisted of 401 DEGs that were downregulated (wtHcp > mutHcp) and 816 that were upregulated (mutHcp > wtHcp) in the mutant Hcp (FDR <0.05; Fig 5A, 5G and 5H). For each tissue, downregulated and upregulated genes were analyzed by Over Representation Analysis (ORA) and Gene Set Enrichment Analysis (GSEA) for Gene Ontology Biological Processes (GO: BPs). In the Hcp, downregulated genes affected the BPs corresponding to DNA conformation, chromosome organization, and DNA replication by both methods of analysis (Fig 5C and 5E), which are consistent with the reduced accessibility at 463 loci in this tissue upon loss of *Lhx2* (Fig 4B). In the Ncp, the downregulated genes affected the BPs corresponding to pathways such as Wnt signaling and Hippo signaling (Fig 5D and 5F). The genes that are upregulated upon the loss of *Lhx2* are associated with a common set of BPs in the Ncp and Hcp, such as Neurogenesis, Neuron projection development, and related processes (S4 Fig), which correspond well with the phenotype of precocious neurogenesis upon the loss of *Lhx2* in both tissues [13,32].

**Fig 6:**
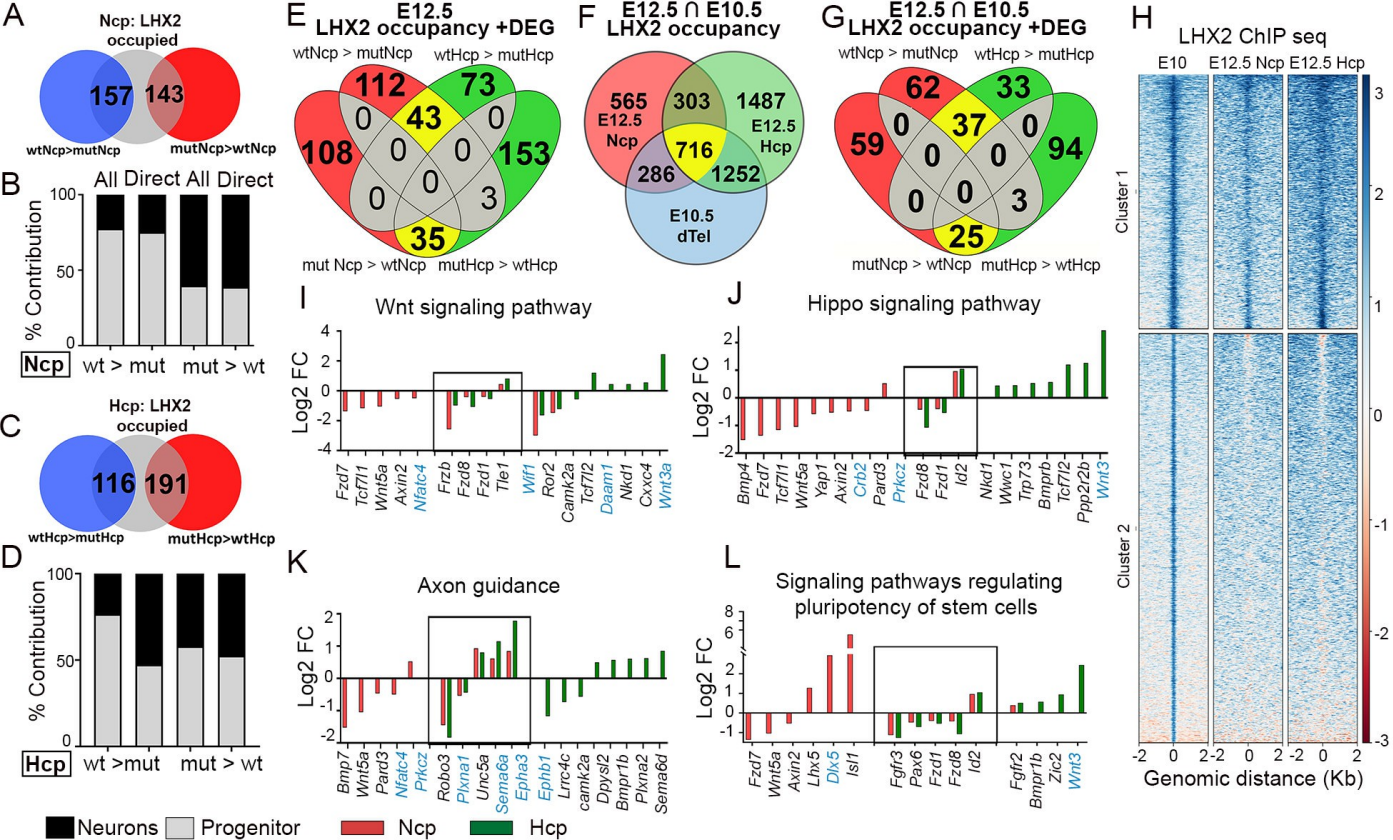
Gene Regulatory Networks modulated by LHX2 in the Ncp and Hcp. (A, C) Venn diagrams depicting the number of genes occupied by LHX2 and dysregulated (blue: downregulated, red: upregulated) upon loss of *Lhx2* in the Ncp (A) and Hcp (C) respectively to identify direct targets of LHX2 in the Ncp and Hcp. (B, D) Genes dysregulated upon loss of *Lhx2* in the Ncp (B) and Hcp (D) respectively, categorized by “Direct” or (direct + indirect) = “All” targets, mapped to the cell-type specific gene enrichment profiles in [28]) to identify progenitor-enriched (grey) and neuron-enriched genes (black). (E) Venn diagram comparing the direct targets of LHX2 that are dysregulated upon loss of *Lhx2* in the Ncp (112 downregulated; 118 upregulated) and Hcp (70 downregulated; 153 upregulated), and in both tissues (43 downregulated; 35 upregulated). (F) Comparison of LHX2 occupancy in the E10.5 dorsal telencephalon (dtel; blue circle) with that in the E12.5 Ncp (red) and Hcp (green) results in genes occupied in all these three tissues (716, yellow), in the E10.5 dtel and the E12.5 Ncp (286, red) or the E12.5 Hcp (1252, green). (G) Venn diagram comparing the genes in E (LHX2 direct targets) that are also occupied by LHX2 at E10.5. In the Ncp, there are 62 downregulated 59 upregulated genes. In the Hcp there are 33 downregulated upregulated 94 upregulated. 37 downregulated and 25 upregulated are common to both tissues. (H) Heatmaps displaying genes occupied by LHX2. Cluster 1: Occupancy at both E10.5 (dtel) and E12.5 (Ncp and Hcp). Cluster 2: Occupancy at only E10.5. (I-L) KEGG pathway analysis (GO: BP) of genes identified in (E, G) reveals 4 pathways dysregulated upon loss of *Lhx2* in the Ncp (red bars) and Hcp (green bars). Individual fold changes are plotted from the RNA-seq data (black: genes occupied by LHX2 at E12.5 and E10.5; blue: occupied only at E12.5).

We compared the genes dysregulated upon loss of *Lhx2* with LHX2 occupancy to arrive at a set of potential direct targets in the Ncp (Fig 6A) and the Hcp (Fig 6C). These “direct” targets as well as “all” DEGs, were then curated using the Ncp scRNA-seq dataset of [28] (Fig 6B and 6D), to identify genes expressed in progenitors (apical/basal) or neurons. In the Ncp, the majority of downregulated DEGs were enriched in progenitors, whereas the majority of upregulated DEGs were enriched in neurons (Fig 6B). These data are consistent with the established role of *Lhx2* in maintaining progenitor proliferation and premature cell-cycle exit and depletion of the progenitor population upon loss of *Lhx2* (Fig 5K, [9,14,32]). Such a pattern was not obvious in the Hcp DEGs. We sought to define the core GRNs regulated by LHX2 in the Ncp and Hcp, first examining only the potential “direct” targets. Comparing potential direct targets of LHX2 in both tissues revealed DEGs unique to the Ncp and the Hcp and some common genes (Fig 6E). Four common signaling pathways emerged from these datasets in both tissues: Wnt signaling, Hippo signaling, Signaling pathways related to pluripotency of stem cells, and Axon guidance (S5A and S5B Fig). In each pathway, there were LHX2 targets unique to each tissue as well as some common targets, the fold changes for which are displayed in Fig 6I–6L). Since these dysregulated pathways are fundamental to developmental processes, we examined whether LHX2 occupied the corresponding genes at E10.5. At this stage, the dorsal telencephalon (dtel) is almost entirely composed of apical progenitors proliferating in self-expansion mode, and the medial primordium that will later form the Hcp has not invaginated substantially. Therefore, we compared LHX2 occupancy in E10.5 dtel tissue (Fig 6H) with the E12.5 Ncp and Hcp occupancy data (Fig 3). Approximately 50% of the E10.5 dtel-occupied genes (2254) overlapped with genes occupied by LHX2 at E12.5. 286 of these overlapped with the E12.5 Ncp, 1252 with the E12.5 Hcp, and 716 were occupied in both primordia (Fig 6F). Several of these genes were present in the four signaling pathways identified to be dysregulated at E12.5 (black gene names, Fig 6I–6L), and only a handful were occupied only at E12.5 (blue gene names, Fig 6I–6L). We extended our analysis to indirect targets of LHX2 that belong to the same KEGG pathways identified for the direct targets, resulting in common as well as Ncp/Hcp-specific GRNs. Interestingly, these indirect targets included several ligands and receptors known to participate in these same four pathways, such as *Rspo1*,*3*, *Wnt2b*, *Fzd3*, *Tgfbr2*, *Bmpr2*, *Slit2*,*3*, *Sfrp4*, and *Cxcl12* (S5 Fig).

**Fig 7:**
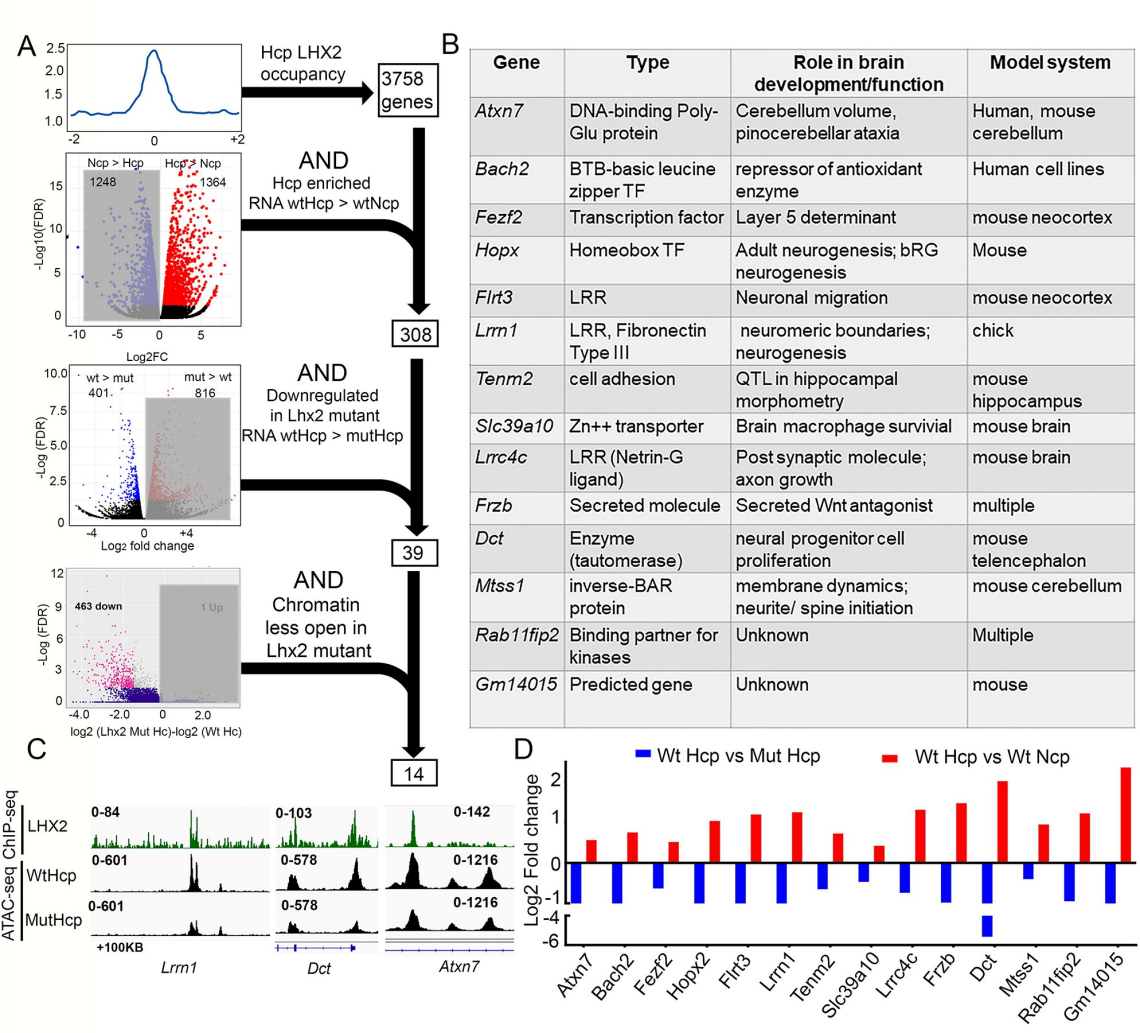
Successive filtration identifies 14 LHX2 target genes relevant to hippocampal development. (A) Four filters were used: First, LHX2 occupancy peaks in the Hcp were mapped to 3758 associated genes. Of these, 308 genes were preferentially expressed in the Hcp (wtNcp< wtHcp). Of these, 39 genes were downregulated upon loss of *Lhx2* (wtHcp>mutHcp). Of these, 14 displayed a decrease in chromatin accessibility upon loss of *Lhx2* (ATAC-seq: wtHcp>mutHcp). (B) A table of the type of protein encoded and known function of these 14 genes. (C) IGV plots for 3 of these genes show an associated LHX2 peak at a region where open chromatin closes upon loss of *Lhx2*. (D) Fold change of the RNA-seq data from comparisons of genes enriched in the Hcp (wtHcp> wtNcp) and their downregulation upon loss of *Lhx2* (wtHcp> mutHcp).

As a final step, we focused on LHX2-regulated genes that were unique to the Hcp across multiple datasets. We progressively filtered data from each approach, beginning with the 3758 genes occupied by LHX2 in the Hcp (Fig 3C and 3E). Of these, 308 were also enriched in the wtHcp (Fig 1B: Hcp > Ncp genes). Of these, 39 genes were downregulated in the Hcp upon loss of *Lhx2* (Fig 5A). Finally, of these, 14 genes displayed decreased chromatin accessibility in the mutHcp (Fig 7A). This group contains genes that encode molecules with established roles in development: DNA-binding protein *Atxn7*; transcription factors *Bach2*, *Fezf2*, *Hopx*; membrane-associated molecules *Flrt3*, *Lrrn1*, *Tenm2*, *Slc39a10*; ligand/secreted molecule *Lrrc4c*, *Frzb*; enzyme *Dct*; cytoplasmic protein *Mtss1*; kinase binding partner *Rab11fip2*, and a previously uncharacterized *Gm14015* (Fig 7B). These 14 genes represent a unique set that is occupied and regulated by LHX2 at the level of chromatin accessibility and mRNA expression in the Hcp (IGV tracks in Figs 4F, 7C and S6D). None of these genes has thus far been examined for a role in hippocampal development and offer new avenues for understanding the ontology of this structure.

## Discussion

This study compares the primordium of the hippocampus with that of the neocortex in terms of chromatin accessibility, gene expression, and gene regulation in response to the loss of pleiotropic transcription factor LHX2. At E12.5, both tissues share similar cellular compositions, with apical progenitors lining the ventricular zone, basal progenitors above them, and newborn neurons and Cajal-Retzius cells residing near the pial surface. Although the extreme lateral portion of the Ncp has accumulated more postmitotic neurons than the dorsal Ncp or the Hcp, this does not account for the large set of DEGs (>1200) that were preferentially enriched in each tissue. The top KEGG pathways arising from the DEGs were largely non-overlapping, with Wnt signaling being the only common one. Furthermore, we found a striking difference in the accessibility of the chromatin, with the Hcp being more accessible at >14000 regions than the Ncp (DARs), (against a background of >100,000 accessible regions in each tissue). The DARs mapped to 9580 genes (S2 Table). Differential accessibility of chromatin in regulatory regions is a key driving factor of patterning and fate specification as shown by studies in the mouse and human telencephalon [24,33–35]. Together, these findings are consistent with the idea that the superficially similar Hcp and Ncp have already embarked on the process of establishing their distinct molecular features.

*Lhx2*, a “cortical selector” gene, is among a small number of fundamental regulators of cell fate in the cortical primordium. Loss of *Foxg1* results in a loss of the entire Ncp, such that all remaining dorsal telencephalic tissue displays hippocampal and Cajal-Retzius cell markers [36]. A complementary phenotype arises from the combined loss of *Emx1* and *Emx2*, in which the Hcp is missing and only the Ncp is present [37]. Both primordia are missing upon loss of *Lhx2*, and the entire dorsal telencephalic neuroepithelium is transformed into either the hem or the antihem, structures that normally flank the Hcp and the Ncp respectively [6]. Thus, loss of *Lhx2* uncovers intrinsic differences within the Ncp and Hcp; therefore, it is not surprising that the LHX2 consensus binding motif appears in the topmost DARs for the two primordia. This fundamental cortical selector role of *Lhx2* impacts both the Ncp and the Hcp, even though *Lhx2* mRNA expression [5] and the total number of LHX2 occupancy peaks are higher in the Hcp (5166) than in the Ncp (2222). In terms of gene dysregulation upon loss of *Lhx2*, there are twice as many DEGs (2372) in the Ncp than in the Hcp (1217), indicating that the relative levels of LHX2 may not necessarily correlate with its function. Intriguingly, chromatin accessibility is altered upon loss of *Lhx2* in 464 regions only in the Hcp. This indicates a major functional difference in terms of how LHX2 regulates the development of the Ncp and the Hcp. Although the loss of *Lhx2* has consequences on cell fate, axon guidance, and neuronal morphology in the Ncp [4], the mechanism of LHX2 action appears not to involve regulating chromatin accessibility in this tissue, suggesting that LHX2 may act in concert with other TFs that perform this role [24,38]. In contrast to the Ncp, 464 regions of Hcp chromatin display decreased accessibility upon loss of *Lhx2*. These regions correspond to 406 genes, 311 of which display LHX2 occupancy, suggesting that a key Hcp-specific function of LHX2 is to maintain open chromatin in a particular set of loci. This is further borne out by the GSEA analysis of genes downregulated upon loss of LHX2 in the Hcp, which maps to pathways related to DNA and chromosome organization.

Our analysis identified several direct targets of LHX2 known to be enriched in either progenitor or newly postmitotic neurons, consistent with LHX2 function in regulating patterning [6,8,39], proliferation [32,40], neuronal subtype identity or properties [11,13], and dendritic arborization [12]. Four major KEGG pathways emerged from the set of all dysregulated genes occupied by LHX2 at E12.5 and some of these were also occupied by LHX2 at E10.5, suggesting that LHX2 control of these pathways begins early and continues into the period of neurogenesis. These pathways are discussed below.

### Hippo pathway

The Hippo signaling pathway regulates overall organ size in several systems [41]. In the brain, it regulates the size of the cortical hem, progenitor proliferation, basal progenitor expansion, synaptic development, corpus callosum formation, and astrocyte differentiation [42,43]. Many of these functions overlap with those reported for *Lhx2*. Loss of *Lhx2* at different stages causes expansion of the hem, shrinkage of the neocortex and hippocampus, decreased progenitor proliferation, loss of the corpus callosum, and premature astrogliogenesis [4]. It is therefore intriguing that LHX2 regulates distinct components of this pathway in the Ncp and Hcp (Ncp: *Yap1*, *Pard3*, *Prkcz*, *Tcf7l1*, *Fzd7*; Hcp: *Wwc1*, *Tcf7l2*, *Nkd1*, *Ppp2rb*) and a few common targets in both tissues (*Fzd1*, *Fzd8*, *and Id2*), suggesting that LHX2 may act via the Hippo pathway for some of its functions.

### Wnt pathway

The Ncp and Hcp are exposed to distinct members of the Wnt family of ligands: the Hcp develops adjacent to the hem which expresses *Wnt2b*, *Wnt3a*, *Wnt5a*, and *Wnt8b*, while the Ncp is exposed to *Wnt4*, *Wnt5a*, *Wnt5b*, *Wnt7a*, *Wnt7b* ([28,44]; this study). In the Ncp, loss of *Lhx2* leads to precocious neurogenesis via the Wnt-β catenin pathway [40]. In the Hcp, it is well-established that canonical Wnt signaling is necessary and sufficient to induce hippocampal fate [45,46]. The cortical hem, a Wnt-rich signaling center, is the hippocampal organizer. Ectopically positioned cortical hems induce adjacent telencephalic neuroepithelium to differentiate into ectopic hippocampi [6]. The mechanisms in the responding tissue that mediate the inductive effects of the hem are unexplored and are likely to be central to the specification and acquisition of hippocampal identity. Our study identifies direct targets of LHX2 in the Ncp and Hcp, which include Wnt ligands and receptors (Figs 6I and S5C). Our identification of distinct (Ncp: *Fzd7*, *Wnt5a*, *Axin2*, *Tcf7l1*, *and Nfatc4;* Hcp: *Wif1*, *Ror2*, *Camk2a*, *Tcf7l2*, *Nkd1*, *Cxxc4*, *Wnt3a)*, as well as common components of the Wnt pathway in the Ncp and Hcp *(Fzd1*, *Fzd8*, *Frzb*, *and Tle1)* as targets of LHX2, offers insight into how the medial primordium may be differentially programmed to execute inductive Wnt signals from the hem.

### Pathways regulating pluripotency and Axon guidance

Loss of *Lhx2* leads to the premature cell-cycle exit of progenitors and precocious neuronal production [32,40]. The genes corresponding to “pluripotency of stem cells” such as *Pax6*, *Fgfr3*, *Fzd1*, and *Fzd8* were downregulated, and, *Id2* was upregulated in both tissues, suggesting that a common GRN governed by LHX2 controls progenitor maintenance in the Ncp and Hcp. As an extension of this, the neurons generated in the mutant are likely to be further along in their differentiation, and hence, the dysregulation of axon guidance pathways may be explained. One established role of LHX2 is in the regulation of dendritic morphogenesis where it directly controls the expression of activity-regulated factor *Btbd3* in postmitotic neurons [12]. Furthermore, loss of *Lhx2* from progenitors at E11.5, which produce subplate cells, results in an exuberant and premature ingrowth of thalamocortical axons into the developing neocortex [11]. The axon guidance targets we identify (Ncp: *Nfatc4*, *Wnt5a*, *Bmp7*; Hcp: *Ephb1*, *Lrrc4c*, *Camk2a*, *Plxna2*, *Sema6d*; both: *Robo3*, *Plxn1a*, *Unc5a*, *Sema6a*) fit well with this role of LHX2 in the broad area of neuronal morphogenesis.

### Crosstalk among pathways

It is not surprising that the target genes we identified appear in multiple pathways, e.g. *Fzd* receptors are included in both Wnt and Hippo signaling KEGG pathways (Fig 6I–6L, [47]). Indeed, crosstalk between these pathways has been investigated in cancer [48], but their interactions in cortical development remain to be fully understood. An intriguing observation was that the majority of the Wnt and Hippo signaling components we identified are downregulated upon loss of *Lhx2* in the Ncp and upregulated in the Hcp. This was a consistent observation whether we examined putative direct or indirect targets of LHX2 (Figs 6 and S5). Combining TF occupancy, accessibility, and transcriptomic dysregulation offers insights into the GRNs that operate in a tissue. Using such an approach, Ypsilanti et al. [24] identified GRNs controlled by PAX6, NR2F1, and EMX2 in cortical patterning. This study also identified regions of combinatorial occupancy between these factors together with LHX2 and PBX1 on particular enhancers that drive expression in discrete regions of the dorsal telencephalic neuroepithelium. Other studies examined the temporal dynamics of cortical development [28,49,50] or compared the developing neocortex with the thalamus [51] or dorsal versus ventral telencephalon [52]. We report the first comparison of the neocortical and hippocampal primordia in which progressive filtering identified 14 genes enriched in the Hcp compared with the Ncp, which depend on LHX2 for maintaining chromatin accessibility as well as mRNA expression. Differential accessibility of chromatin in regulatory regions is a key driving factor of patterning and fate specification, as shown by studies in the mouse and human telencephalon [24,33–35]. In this context, our findings offer a rich dataset for further analysis of the mechanisms that distinguish the Hcp from the Ncp and promote the distinctive development of these structures.

## Methods

### Ethics statement

All animal protocols were approved by the Institutional Animal Ethics Committee of the Tata Institute of Fundamental Research (TIFR-IAEC) which ensures that all animal studies were conducted in accordance with ethical guidelines.

### Mice

The floxed LIM homeobox2 (*Lhx2*) line (*Lhx2lox/lox*) and *Emx1CreYL* lines used in this study have been described previously by [6,29]. The *Emx1CreYL* [29] was obtained as a gift from Prof. Yuqing Li at the University of Florida College of Medicine. The floxed *Lhx2* line was a gift from Prof. Edwin Monuki at the University of California, Irvine. Timed pregnant female mice were obtained from the Tata Institute animal breeding facility, and embryos of both sexes were used for the experiments, with the *Emx1CreYL* contributed from the male parent. Noon of the day the vaginal plug was observed was considered E0.5. Early-age embryos were staged by somite number, genotyped using PCR and assigned to groups accordingly. Controls used for each experiment were age-matched littermates. The mT/mG reporter mouse line was obtained from JAX labs Stock No. 007576; this reporter was used to check for cre activity in the brain. All animals were kept at an ambient temperature and humidity, with a 12 hr. light-dark cycle and food available ad libitum. Primers used for genotyping were: Cre F: 5′ATTTGCCTGCATTACCGGTC3′, Cre R: 5′ATCAACGTTTTCTTTTCGG3′, Cre-positive DNA shows a band at 350 bp. *Lhx2* cKO forward: 5’ACCGGTGGAGGAAGACTTTT3’, *Lhx2* cKO reverse: 5’CAGCGGTTAAGTATTGGGACA3’. The band sizes for this PCR are as follows: Wild-type: 144 bp, *Lhx2Cko*: 188 bp.

### ATAC-seq and data analysis

ATAC-seq was performed using 2 biological replicates of each sample (wtNcp, wtHcp, mutNcp, mutHcp). The tissue was collected in ice-cold PBS containing 0.5% glucose and was triturated using a Dounce homogenizer to obtain a single-cell suspension. The number of live cells was counted on a hemocytometer using trypan blue to stain dead cells. 50,000 cells were used for each n, and 3n’s each were processed for Omni-ATAC seq with modification from Amanda Ackermann’s lab. Briefly, cells were washed with 1x DPBS and re-suspended in cell lysis buffer (10 mM Tris pH 7.5, 10 mM NaCl, 3 mM KCl, 0.1% NP-40, 0.1% Tween20 and 0.01% Digitonin) and incubated on ice for 3 minutes. Further, cells were washed with a wash buffer (10 mM Tris pH 7.5, 10 mM NaCl, 3 mM KCl and 0.1% Tween20) by centrifugation at 500g for 10 minutes at 4°C. The supernatant was discarded, and the pellet was resuspended in 25 μl 2x Tagmentation buffer (Illumina, catalog #15027866), 16.5 μl DPBS, 0.5 μl 10% Tween 20, 0.5 μl 1% Digitonin, 5 μl nuclease and 2.5 μl Tn5 transposase enzyme (TDE1, Illumina, catalog # 15027865) and incubated for 28 minutes at 37°C. After the tagmentation reaction, DNA was isolated using the Zymo DNA clean and concentrator kit (Zymo). Purified DNA was used as an input to generate a library by amplifying with 2x Q5 DNA polymerase mix (NEB) and indexing primers. Optimal cycles were determined using qPCR analysis. Amplified libraries were purified using Agencourt ampure XP beads to remove adapters and larger fragments.

Sequencing reads (41 bp PE) were obtained on NextSeq-550 at IISER Pune and trimmed for Nextera adapters using default parameters of Trimmomatic PE. Trimmed reads were aligned to mm10 using the default parameters of Bowtie2 [53]. Briefly, BAM files were subsampled to 50 million reads in each sample using BBMap and sorted by name. Paired-end bed files were obtained using the bamtobed function of bedtools. Reads were displaced by +4 bp and -5 bp. Peak calling was performed using MACS2 callpeak -f BEDPE -q 0.05—nomodel—extsize 200—gsize 1.3e9—keep-dup 2 parameters. Consensus peaks were obtained using a custom R script used for ChIP-seq analysis. BigWig files were generated using bamCoverage (deepTools). Peaks were annotated to the nearest gene using Homer and classified into promoter (+/- 2 Kb) and non-promoter regions based on Homer annotatePeaks.pl function ([54], http://homer.ucsd.edu/homer/ngs/quantification.html). K-means clustering was performed around +/- 2 Kb of LHX2 peak center using deepTools. Motif analysis was performed using findMotifsGenome.pl from Homer. LHX2-occupied DARs were overlapping by at least 1 bp were identified using Bedtools and annotated using Homer function (annotatePeaks.pl).

### Differential chromatin accessibility analysis (Diffbind)

The differential chromatin accessibility analysis was performed using DiffBind [55]. Significantly differentially accessible peaks were identified using the DESeq2 package and only sites with FDR < 0.05 and fold change of > Log2 (+/- 1.5) were used for further analysis. Differentially accessible sites were annotated to the nearest gene using Homer. The core promoter was defined as +/- 2 Kb from the TSS.

### Genome-wide occupancy analysis (ChIP-seq and data analysis)

LHX2 ChIP-seq was performed in 4 biological replicates of each sample (E12.5 Ncp, E12.5 Hcp, E10.5 dorsal telencephalon). ChIP-seq for histone marks was performed using a single sample to generate 2 technical replicates, and the fastq files were combined to generate a single file and processed downstream. Input DNA was used as a control and sequenced with the respective samples for all ChIP-seq experiments.

### Tissue processing

Neocortical and hippocampal tissue were dissected from E12.5 Swiss mice and collected in ice-cold PBS containing 0.5% glucose and a protease inhibitor cocktail (P8340). Tissue was cross-linked using 1% formaldehyde (#47608) for 8 minutes, followed by quenching with 125 mM glycine for 5 minutes at RT. The chromatin was sheared using a focused sonicator (Covaris) to obtain fragments of 100–300 bp. 100 μg (for LHX2) or 60 μg (for Histone marks) of sheared chromatin was used to set up an IP and 10% of the chromatin volume was stored as input. Dynabeads A and G were mixed in a 1:1 ratio and used to pull down the antibody-protein complex. Beads were washed 3 times with low salt buffer (20 mM Tris HCl pH 8.0, 150 mM NaCl, 2 mM EDTA, 0.1% SDS, 1% Triton X-100), followed by 2 washes with high salt buffer (20 mM Tris HCl pH 8.0, 200 mM NaCl, 2 mM EDTA, 0.1% SDS, 1% Triton X-100), 1 wash with LiCl buffer (0.25 M LiCl, 1 mM EDTA, 10 mM Tris HCl pH 8.0, 1% NP-40, 1% sodium deoxycholate) and 2 washes with TE buffer (10 mM Tris HCl pH 8.0, 1 mM EDTA). The beads were resuspended in 150 μl of elution buffer (0.1M NaHCO_3_, 1% SDS) and at 65°C for 30 minutes at 1000 rpm. The eluate was collected in fresh tubes and the elution was repeated to obtain a total eluate of 300 μl. The IP and input samples were reverse cross-linked using 20 μL of 5 M NaCl and 2 μL of RNAseA (10 mg/ml), and incubated overnight at 65°C at 800 rpm. The samples were then treated with 20 μL of 1 M Tris pH 8.0, 10 μL of 0.5 M EDTA and 2 μL of Proteinase K (20 mg/ml) and incubated at 42°C for 1 hr at 800 rpm. Samples were purified using phenol: chloroform: isoamyl alcohol and DNA was precipitated at -20°C using 2X volume of 100% ethanol, 100 mM sodium acetate and Glycoblue (#AM9515). DNA pellets were resuspended in nuclease-free water and quantified using a Qubit fluorometer (Thermo Fisher Scientific, USA) for downstream processing.

### Antibodies

LHX2 antibody was from Santa Cruz Biotechnology (Sc-19344). For Histone ChIP-seq, the following antibodies were used: H3K4Me1 (C15310037, Diagenode), H3K4Me3 (ab8580, Abcam), H3K2Ac (ab4729, Abcam) and H3K27Me3 (07–449, Millipore).

### Library preparation, sequencing and data analysis

An equal amount of DNA (~5–8 ng) was used as an input for library preparation and libraries were prepared using an NEB Ultra II DNA library prep kit (NEB, USA). Sequencing reads (100 bp PE) were obtained on the HiseqX platform at Macrogen, Korea.

Sequencing reads were trimmed using TrimmomaticPE for Truseq2:PE adapters and were aligned to the mouse mm10 genome using the default parameters of BWA. Aligned reads were subsampled to 25 million reads for each sample using BBMap. For the LHX2 ChIP-seq the QC, peak calling was performed using default parameters in PePr. Only statistically significant peaks were used for further analysis (p-value 0.0001 and fold change over input: cut off >10 fold). For the Histone ChIP-seq samples, the fastq files were aligned using bowtie. Peak and differential peak-calling and subsequent annotations were done using HOMER. Peaks were annotated to the nearest gene using Homer and classified by Homer using the annotatePeaks.pl function ([54]http://homer.ucsd.edu/homer/ngs/quantification.html).

### RNA sequencing, library preparation, and analysis

The mouse Ncp and Hcp were dissected from E12.5 control and *Lhx2* mutant brains and stored in TrizolⓇ reagent. Tissue dissected from 4 embryos was pooled to obtain 5 μg RNA for each of the 3 biological replicates. After library preparation, sequencing was performed on the Illumina platform to achieve 150 bp reads to generate 30 Million paired-end reads per sample. FastQC was performed as described in (https://www.bioinformatics.babraham.ac.uk/projects/fastqc/), and reads > 30 Phred scores were aligned using HISAT2 [53]. Feature counts were used to quantify the number of reads per transcript. Differential expression analysis was performed using EdgeR [56,57] on the R platform (v3.4.0). Genes showing |log2 Fold change | ≥0.35 and FDR < 0.05 were used for further analysis. Gene ontology analysis was performed using WebGestalt or gShinyGO 0.76 [58,59]. Bar plots were created with GraphPad Prism V9.1.0. Gene-based heatmaps were plotted using normalized reads on Morpheus (Morpheus, https://software.broadinstitute.org/morpheus). Venn diagrams were initially generated using Venny 2.1.0 (https://bioinfogp.cnb.csic.es/tools/venny/) and edited using Adobe Photoshop (CC 2017).

### Immunofluorescence

Mouse brain sections were mounted on plus slides (Catalog number: EMS 71869–11) and dried for 2–3 h. Slides were transferred to a slide mailer (Catalog number: EMS 71549–08) containing PB + 0.1% TritonX-100 for 10 min followed by a wash with PBS + 0.3% TritonX-100 for 5 min. For antigen retrieval, sections were incubated in a 10 mM sodium citrate buffer (pH  =  6) at 90°C for 10 min using a water bath. Slides washed with PBS + 0.01% TritonX-100 for 10 min. Blocking (5% horse serum in PBS + 0.3% TritonX-100) for 1 h followed by overnight primary antibody incubation at 4°C. Secondary antibody incubation was performed at room temperature for 2 h followed by three washes with 1x PB. Slides were mounted using Fluoroshield mounting media (sigma F6182) and imaged in an Olympus FluoView 1200 confocal microscope. The primary antibodies used were: PAX6 (Rabbit, 1:500 Abcam catalog # ab195045), FOXG1(Rabbit 1:200,Takara Catalog #M227), LHX2 (Rabbit, 1;200, Merck catalog #ABE1402), SOX2 (Mouse, 1:200 Invitrogen, #MA1014). Secondary antibodies used were the following: Goat Anti Rabbit Alexa fluor 488 (1:200, Invitrogen catalog # A11034), Goat Antimouse Alexa fluor 594 (1:200, Invitrogen catalog # R37121), Goat Anti Rabbit Alexa fluor 568 (1:200, Invitrogen catalog # A11011), Donkey Antirabbit Alexa fluor 647 (1:200, Invitrogen catalog # A31573).

### RNA-In Situ hybridizations (ISH)

RNA ISH was performed as previously described in [14]. Plasmids used for generating probes were obtained from Francois Guillemot, Crick Institute (*Neurog1* & *Neurog2*); Anastassia Stoykova, University of Göttingen (*Id2*); Robert Hevner, University of Washington (*Tbr1*); Pierre Vanderhagen, KU Leuven (*EphB1*), Cliff Ragsdale, University of Chicago (*Lef1*), Yasushi Nakagawa, University of Minnesota (Lhx9), Jeffrey Macklis, Harvard University (*Nr2f1/CoupTF1*), Ryoichiro Kageyama, Kyoto University (*Hes1*, *Hes6*).

## Supporting information

S1 Fig (A) Bar plot of the distance from TSS of the DARs identified in Fig 2B. (B) 4-quadrant graph of ATAC-seq and RNA-seq fold changes for wtHcp vs wtNcp data from Figs 1B and 2B. 710 genes were identified as Hcp enriched and 19 genes were as Ncp enriched in terms of both open chromatin and RNA expression.(C) Heat maps and cumulative profiles of 14804 (Hcp) and 70 (Ncp) DARs identified in Fig 2B. (D) *IGV tracks of ATAC-seq*, *H3K27Ac*, *H3K4Me3*, *and H3K4Me1*, *together with input tracks for Ncp (red) and Hcp (green)*. *Black boxes mark regions enriched in open chromatin in the Hcp/Ncp that align with one or more histone modifications*. *The numbers on the tracks indicate the maximum peak height*.
(TIF)

S2 Fig (A, B) KEGG pathway analysis of Ncp and Hcp enriched genes related to Fig 1B. (C-D) Motif analysis shows known motifs from 70 DARs (Ncp) and 14804 DARs (Hcp) related to Fig 2B. (E) Expression of many of the transcription factors identified among the top 10 motifs is undetectable in the E11.5 Ncp or Hcp (as obtained from; Allen Mouse Brain Atlas, http://mouse.brain-map.org/). Links to images represented in E: *Dlx1 Dlx2 Dlx5 Lhx1 Lhx3 Isl1 Nkx6.1 En1 Rfx2 Rfx5 Xbp1*.
(TIF)

S3 Fig Characterization of Lhx2 occupancy across genomic regions. (A, B) Lhx2 occupancy profiles in the Ncp and Hcp using Ncp peaks as a reference (A; 2222 peaks); using Hcp peaks as a reference (B, 5166 peaks). (C) Bedtools intersect analysis reveals 688 Lhx2 peaks overlap by at least 1bp in the Lhx2 Ncp (total 2222 peaks) and Hcp (total 5166 peaks) ChIP-seq data.(D) Bar plots of the average gene expression of the Lhx2 occupied genes in the Ncp and Hcp compared with the respective library averages.(E, F) bar plots of the distances of LHX2 peaks from the TSS in the Hcp and Ncp.(G) LHX2 occupancy profiles on 14874 DARs (70 Ncp + 14804 Hcp) from Fig 2B shows multiple DARs occupied by LHX2 in both tissues. (H) IGV tracks showing LHX2 peaks in the Hcp and Ncp together with their respective input control tracks. Black boxes mark regions equally enriched for LHX2 occupancy in Ncp and Hcp; green boxes indicate regions with greater LHX2 occupancy in Hcp; blue boxes indicate regions with greater LHX2 occupancy in Ncp. The numbers on the tracks indicate the maximum peak height. (I) Summary findings of the 360 DARs that mapped to LHX2 occupied regions.
(TIF)

S4 Fig (A-F) GO: BPs corresponding to both up-and down-regulated genes upon loss of *Lhx2* in the Ncp (A-C) and Hcp (D-F). (A, D) show the GSEA analysis and (B, C, E, F) show the overrepresentation test analysis.
(TIF)

S5 Fig (A, B) KEGG pathway analysis of direct targets of LHX2 from Fig 5 (F) reveal 4 dysregulated pathways common to the E12.5 Ncp and Hcp. (C-F) KEGG pathway analysis for these pathways includes both direct (*) and indirect targets of LHX2. (E, F).
(TIF)

S6 Fig (A-C) ChromHMM analysis of the ATAC-seq (mutant and wt), LHX2 ChIP-seq, and histone ChIP-seq of H3K27Ac, H3K4Me3, H3K4Me1 and H3K27Me3 in the wtNcp (A), wtHcp (B) and mutHcp (C). In each column the first row of ChromHMM profiles shows the emission parameters used and the state (emission order) for all the samples used to generate the matrix. The rows 2–4 in (A-C) identify regions of the genome enriched for various samples and their corresponding emission. (D) IGV tracks of LHX2 Hcp occupancy and ATAC-seq peaks for wtHcp and mutHcp. Black boxes mark LHX2 occupied regions that show a decrease of open chromatin in the mutHcp. The numbers on the tracks indicate the maximum peak height used to generate tracks.
(TIF)

S1 Table Worksheet containing DEGs identified in Fig 1B, lists of TF DEGs, wtNcp>wtHcp (111) and wtHcp>wtNcp (94). (XLSX)

S2 Table Worksheet containing raw data of DARs identified in Fig 2B, a list of annotated DARs identified in Fig 2B, a comparative analysis of genes associated with DARs and gene expression in wtHcp vs wtNcp related to S1B Fig. (XLSX)

S3 Table Worksheets containing LHX2 peaks from E12.5 Ncp, E12.5 Hcp and E10.5 dtel. A list that identifies LHX2 peaks that overlap between E12.5 Ncp and E12.5 Hcp. A list identifies expression levels (in CPM) of all genes associated with one or more LHX2 peaks in the Hcp and Ncp.
(XLSX)

S4 Table Worksheets containing raw data of DARs identified in Fig 4B, a list of annotated DARs identified in Fig 4B, an intersection list of DAR-associated genes that are also occupied by LHX2 in the wtHcp (311 DAGs) identified in Fig 4E. (XLSX)

S5 Table Worksheets containing raw data of identifying DEGs of mutNcp vs wtNcp Fig 5A, DEGs of mutHcp vs wtHcp in Fig 5B, a list of all genes identified by Venn diagram in Fig 6E. (XLSX)

S1 Data Worksheets containing differential peaks called and annotated by Homer in the wtNcp vs input for histone modifications H3k27Ac, H3k27Me3, H3k4Me3, and H3k4Me1. (ZIP)

S2 Data Worksheets containing differential peaks called and annotated by Homer in the wtHcp vs input for histone modifications H3k27Ac, H3k27Me3, H3k4Me3, and H3k4Me1. (ZIP)
