# Supplementary figures and images for: Regulation of chromatin accessibility and gene expression in the developing hippocampal primordium by LIM-HD transcription factor LHX2
Source: PLoS Genet. 2023 Aug 18;19(8):e1010874. doi: 10.1371/journal.pgen.1010874 (PMC10482279; doi:10.1371/journal.pgen.1010874)

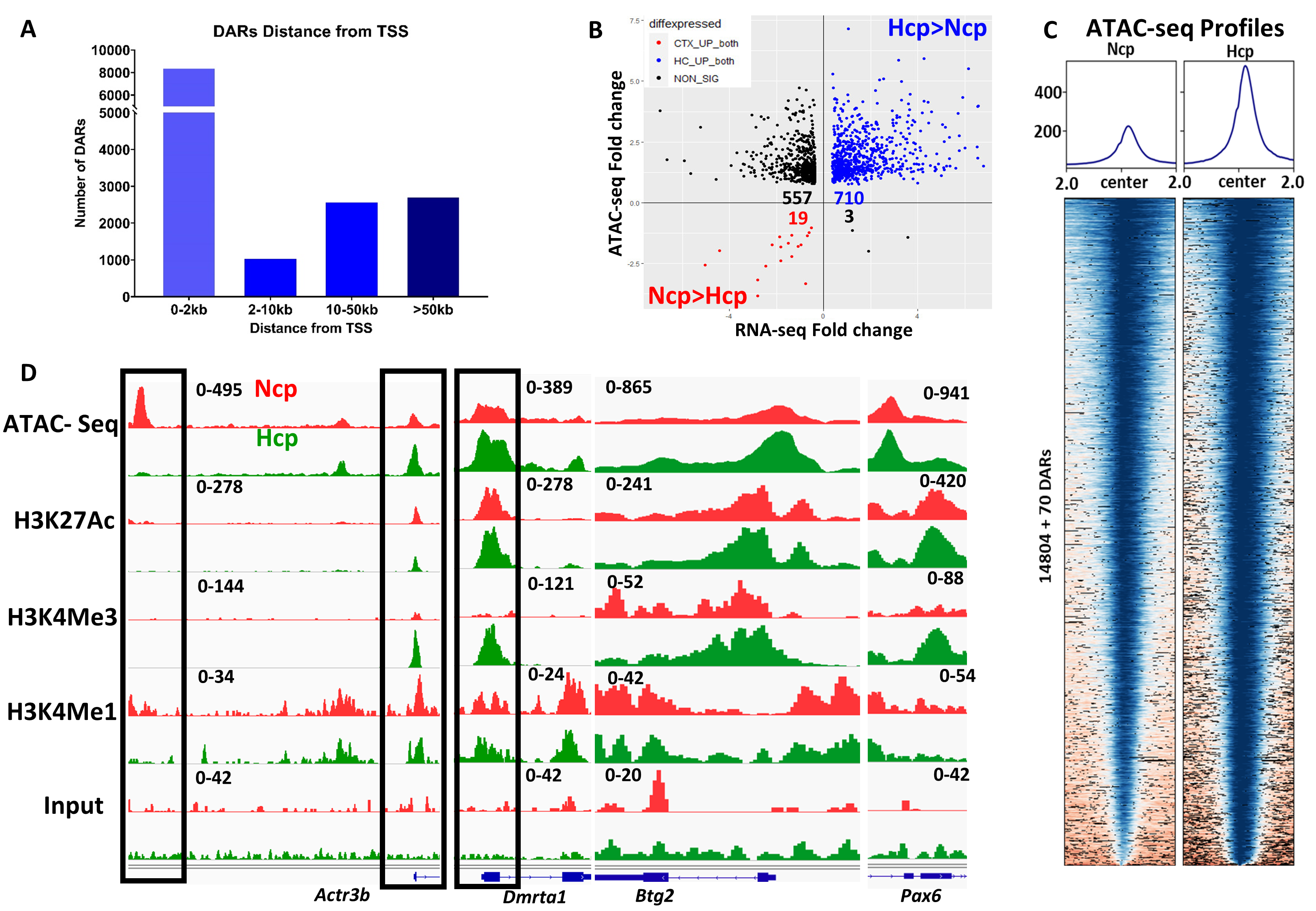

Supplement: S1 Fig — (A) Bar plot of the distance from TSS of the DARs identified in Fig 2B. (B) 4-quadrant graph of ATAC-seq and RNA-seq fold changes for wtHcp vs wtNcp data from Figs 1B and 2B. 710 genes were identified as Hcp enriched and 19 genes were as Ncp enriched in terms of both open chromatin and RNA expression.(C) Heat maps and cumulative profiles of 14804 (Hcp) and 70 (Ncp) DARs identified in Fig 2B. (D) IGV tracks of ATAC-seq, H3K27Ac, H3K4Me3, and H3K4Me1, together with input tracks for Ncp (red) and Hcp (green). Black boxes mark regions enriched in open chromatin in the Hcp/Ncp that align with one or more histone modifications. The numbers on the tracks indicate the maximum peak height. (TIF) [file pgen.1010874.s001.tif]

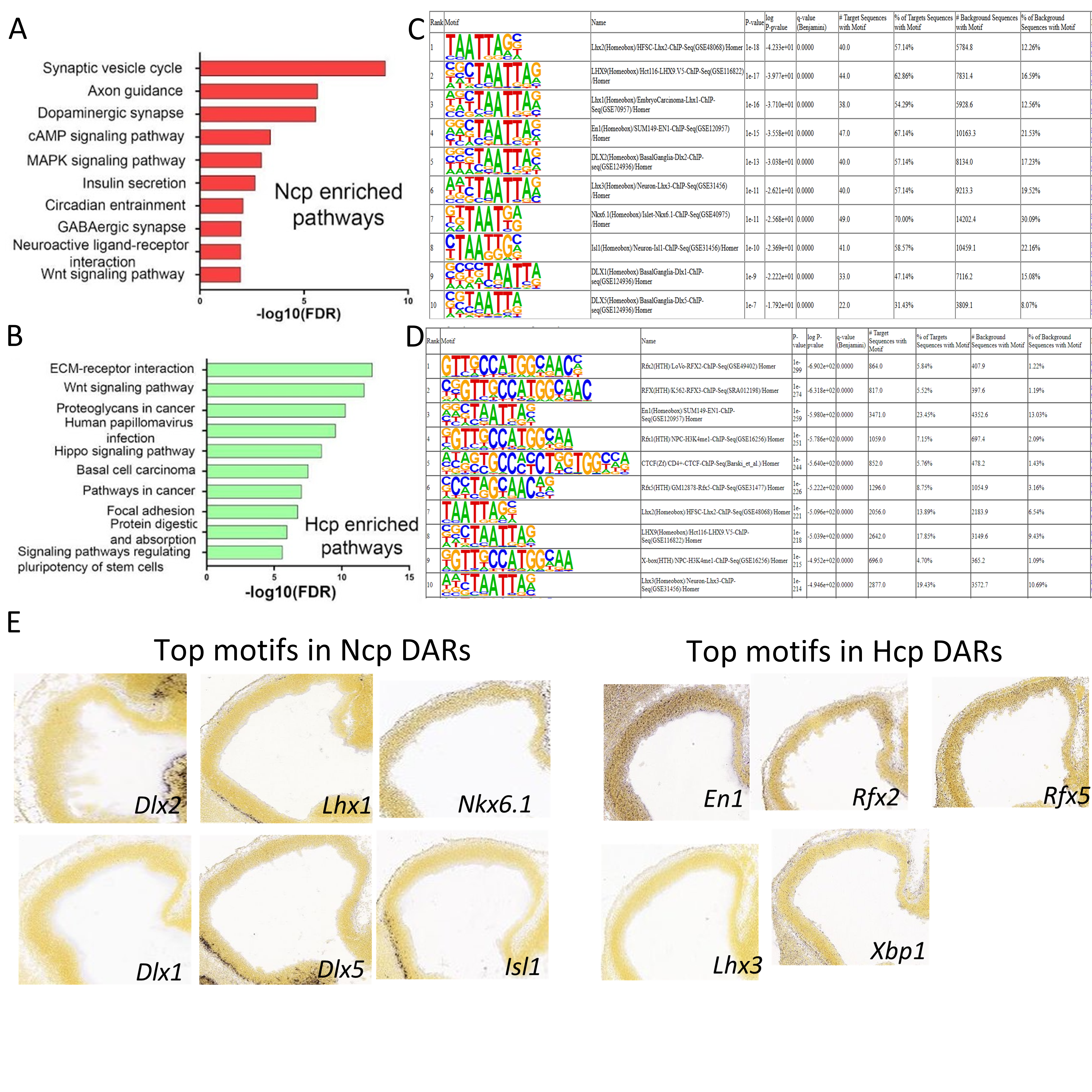

Supplement: S2 Fig — (A, B) KEGG pathway analysis of Ncp and Hcp enriched genes related to Fig 1B. (C-D) Motif analysis shows known motifs from 70 DARs (Ncp) and 14804 DARs (Hcp) related to Fig 2B. (E) Expression of many of the transcription factors identified among the top 10 motifs is undetectable in the E11.5 Ncp or Hcp (as obtained from; Allen Mouse Brain Atlas, http://mouse.brain-map.org/). Links to images represented in E: Dlx1 Dlx2 Dlx5 Lhx1 Lhx3 Isl1 Nkx6.1 En1 Rfx2 Rfx5 Xbp1. (TIF) [file pgen.1010874.s002.tif]

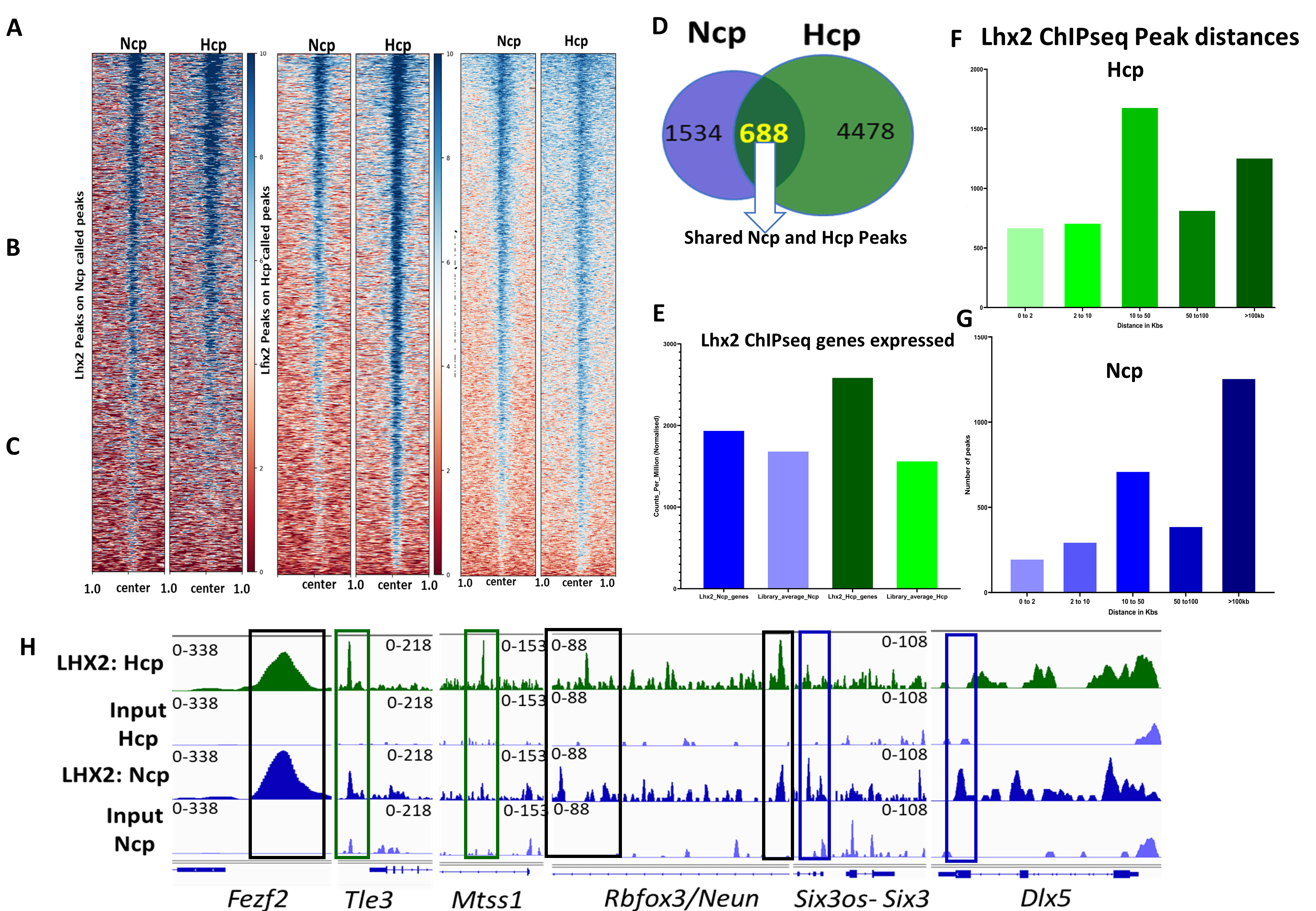

Supplement: S3 Fig — (A, B) Lhx2 occupancy profiles in the Ncp and Hcp using Ncp peaks as a reference (A; 2222 peaks); using Hcp peaks as a reference (B, 5166 peaks). (C) Bedtools intersect analysis reveals 688 Lhx2 peaks overlap by at least 1bp in the Lhx2 Ncp (total 2222 peaks) and Hcp (total 5166 peaks) ChIP-seq data.(D) Bar plots of the average gene expression of the Lhx2 occupied genes in the Ncp and Hcp compared with the respective library averages.(E, F) bar plots of the distances of LHX2 peaks from the TSS in the Hcp and Ncp.(G) LHX2 occupancy profiles on 14874 DARs (70 Ncp + 14804 Hcp) from Fig 2B shows multiple DARs occupied by LHX2 in both tissues. (H) IGV tracks showing LHX2 peaks in the Hcp and Ncp together with their respective input control tracks. Black boxes mark regions equally enriched for LHX2 occupancy in Ncp and Hcp; green boxes indicate regions with greater LHX2 occupancy in Hcp; blue boxes indicate regions with greater LHX2 occupancy in Ncp. The numbers on the tracks indicate the maximum peak height. (I) Summary findings of the 360 DARs that mapped to LHX2 occupied regions. (TIF) [file pgen.1010874.s003.tif]

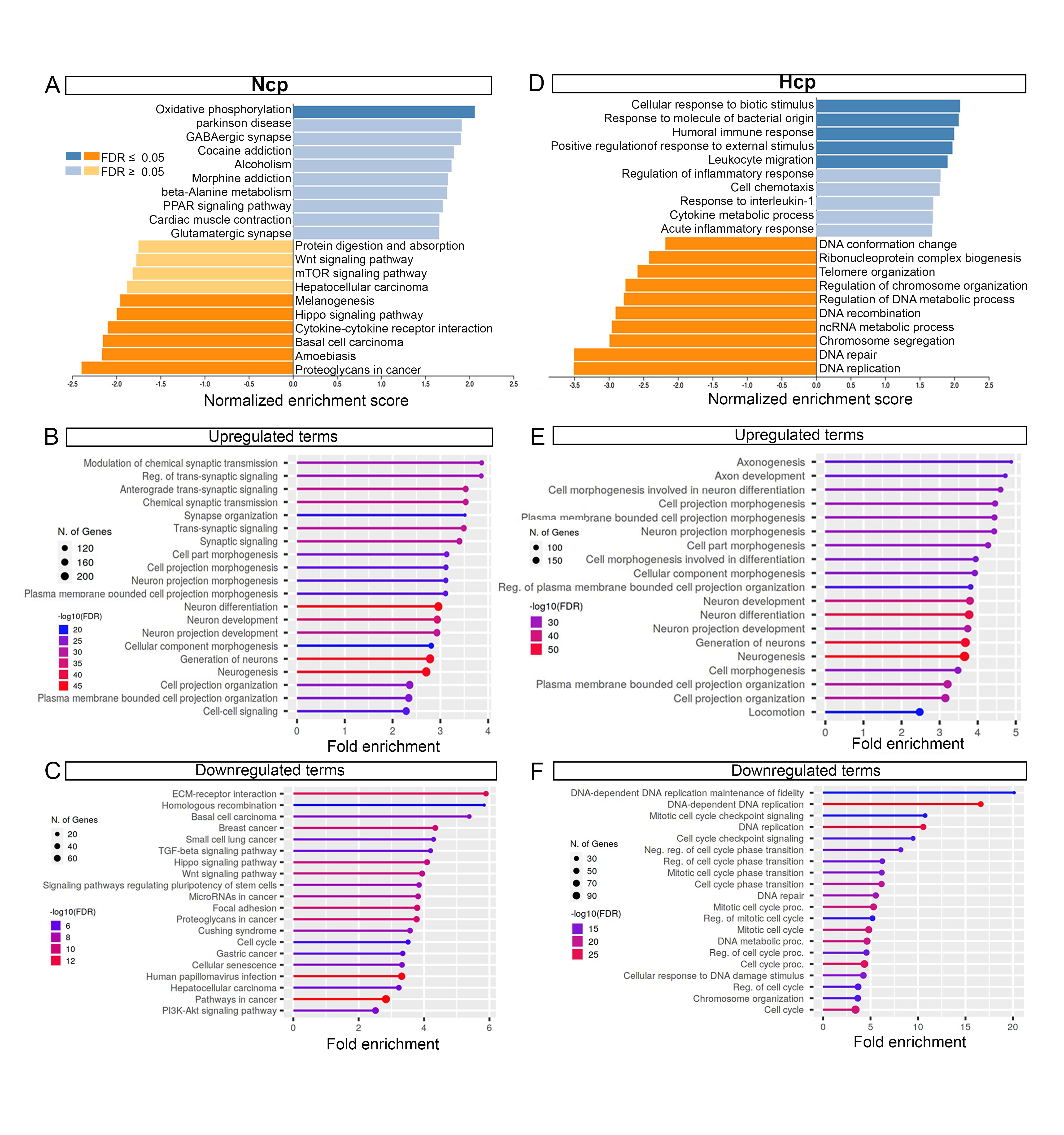

Supplement: S4 Fig — (A-F) GO: BPs corresponding to both up-and down-regulated genes upon loss of Lhx2 in the Ncp (A-C) and Hcp (D-F). (A, D) show the GSEA analysis and (B, C, E, F) show the overrepresentation test analysis. (TIF) [file pgen.1010874.s004.tif]

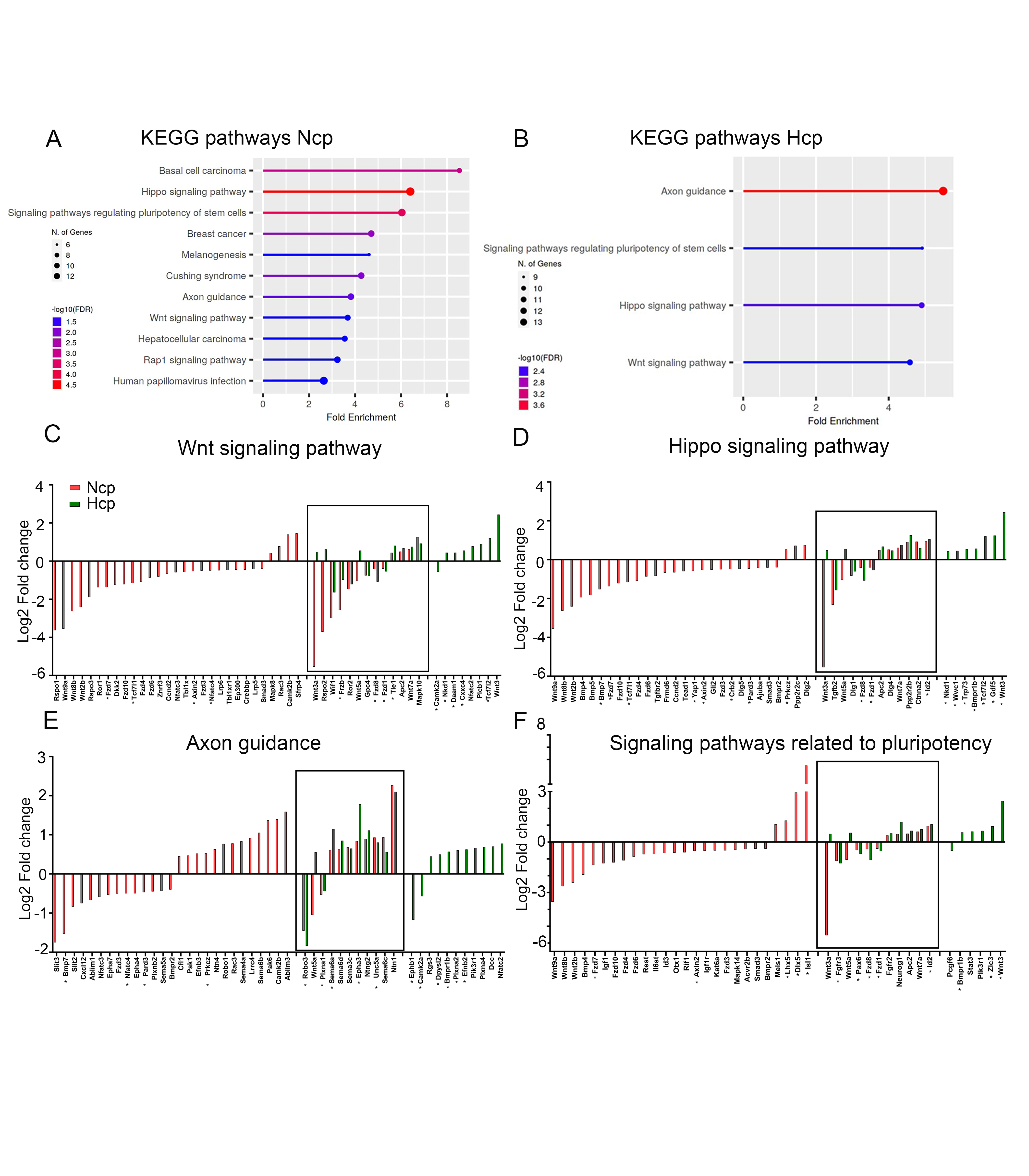

Supplement: S5 Fig — (A, B) KEGG pathway analysis of direct targets of LHX2 from Fig 5 (F) reveal 4 dysregulated pathways common to the E12.5 Ncp and Hcp. (C-F) KEGG pathway analysis for these pathways includes both direct (*) and indirect targets of LHX2. (E, F). (TIF) [file pgen.1010874.s005.tif]

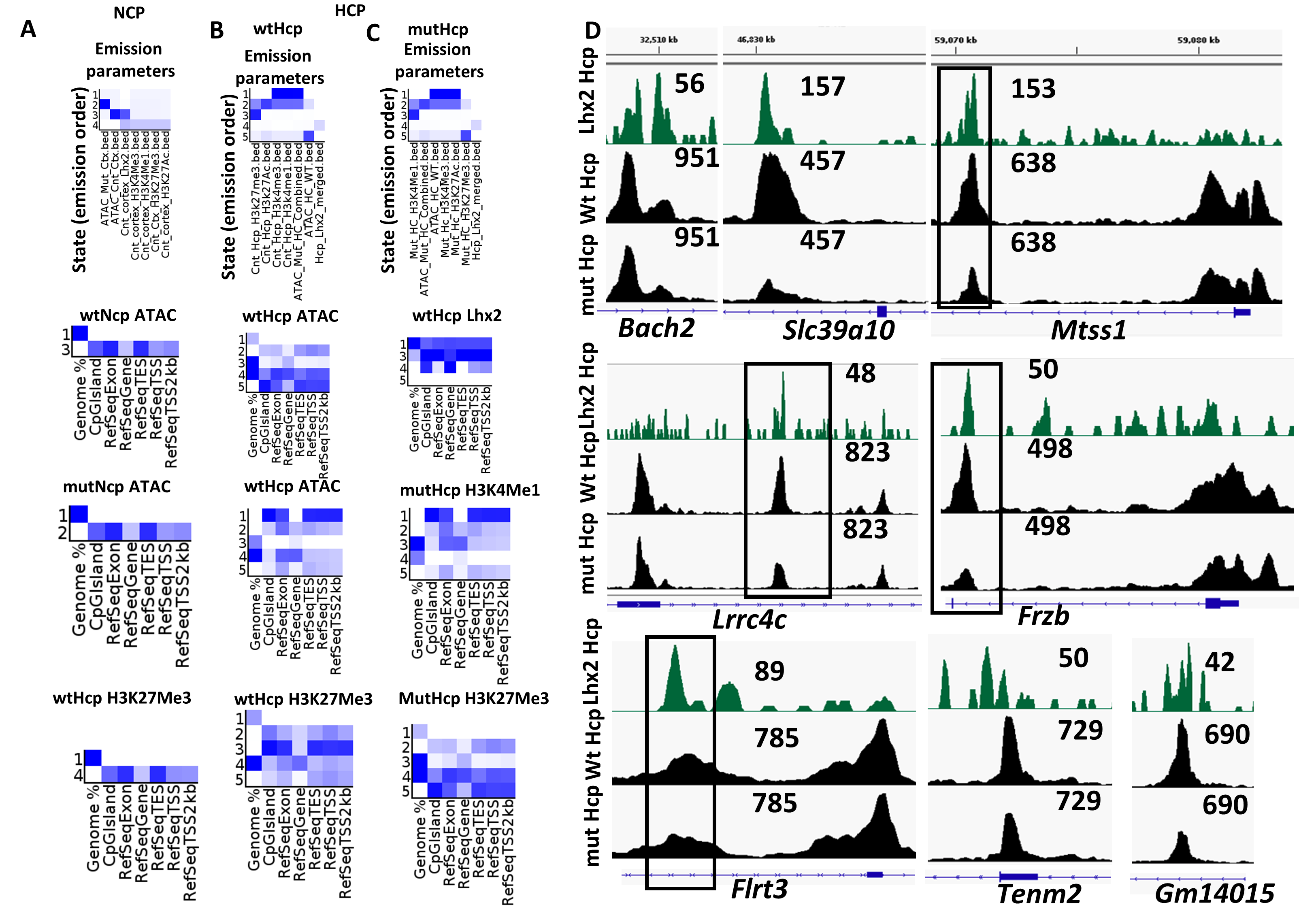

Supplement: S6 Fig — (A-C) ChromHMM analysis of the ATAC-seq (mutant and wt), LHX2 ChIP-seq, and histone ChIP-seq of H3K27Ac, H3K4Me3, H3K4Me1 and H3K27Me3 in the wtNcp (A), wtHcp (B) and mutHcp (C). In each column the first row of ChromHMM profiles shows the emission parameters used and the state (emission order) for all the samples used to generate the matrix. The rows 2–4 in (A-C) identify regions of the genome enriched for various samples and their corresponding emission. (D) IGV tracks of LHX2 Hcp occupancy and ATAC-seq peaks for wtHcp and mutHcp. Black boxes mark LHX2 occupied regions that show a decrease of open chromatin in the mutHcp. The numbers on the tracks indicate the maximum peak height used to generate tracks. (TIF) [file pgen.1010874.s006.tif]
